# Supplementary figures and images for: The ORP9-ORP11 dimer promotes sphingomyelin synthesis
Source: eLife. 2024 Aug 6;12:RP91345. doi: 10.7554/eLife.91345 (PMC11302984; doi:10.7554/eLife.91345)

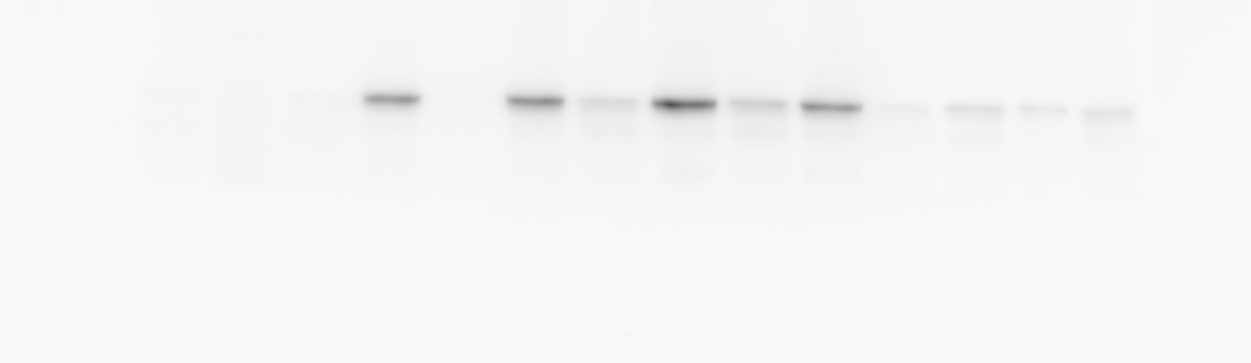

Supplement: Figure 1—source data 1. [file elife-91345-fig1-data1.zip › Figure 1-raw images/Figure 1-source data 11-Raw Images_ORP1L.tif]

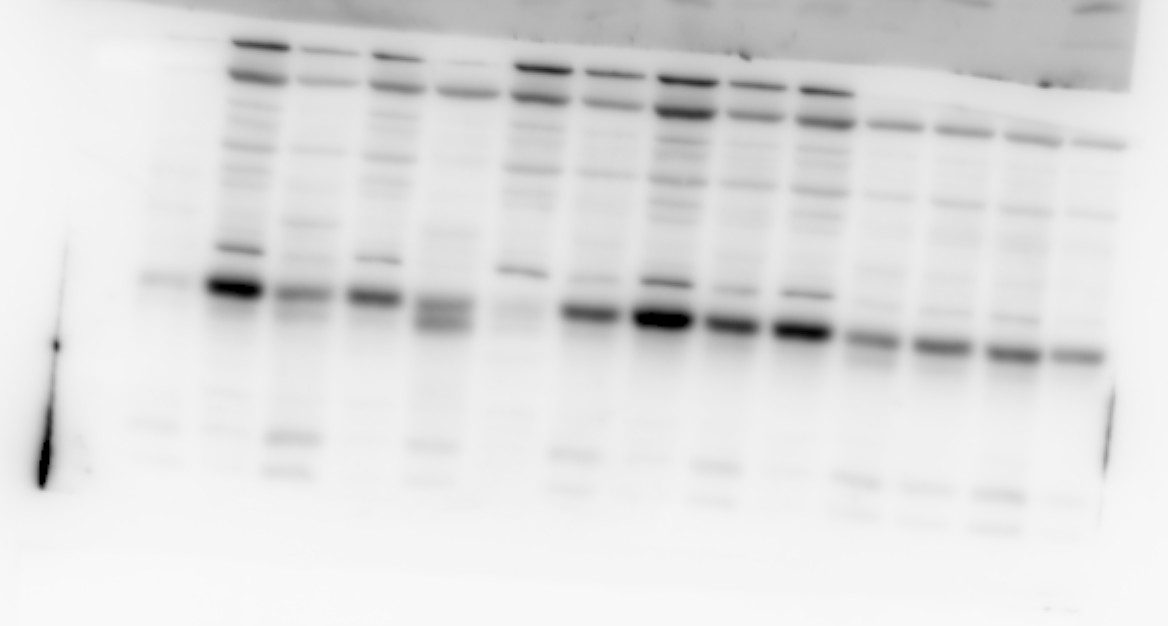

Supplement: Figure 1—source data 1. [file elife-91345-fig1-data1.zip › Figure 1-raw images/Figure 1-source data 25-Raw Images_STARD7.tif]

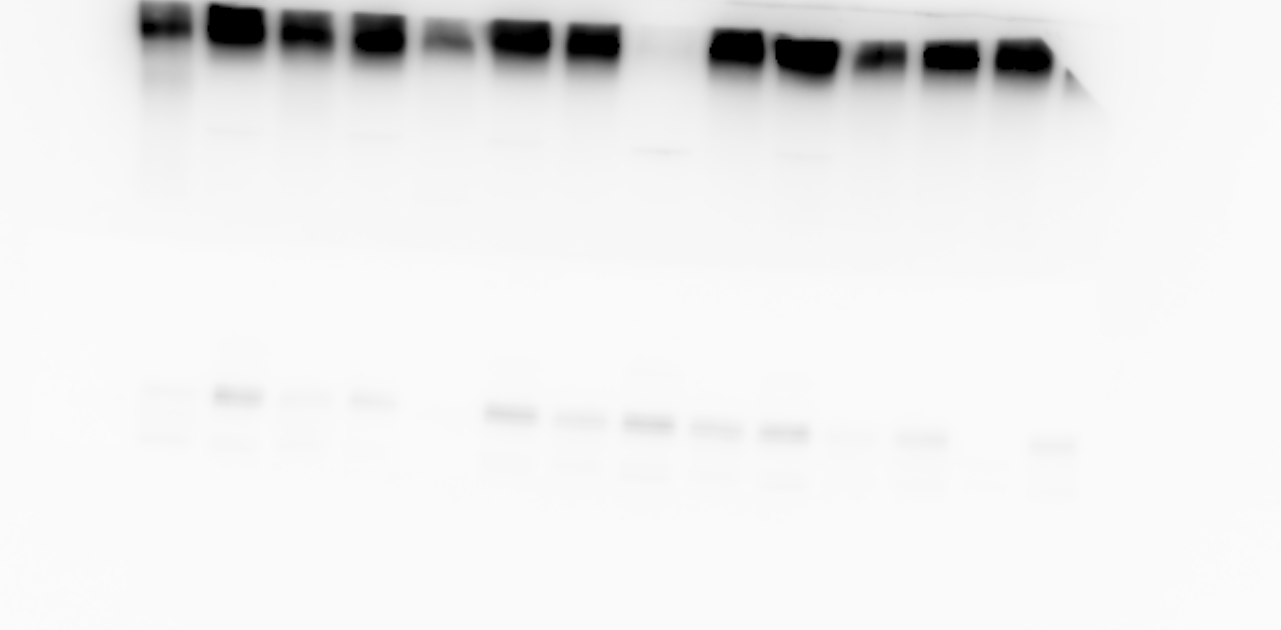

Supplement: Figure 1—source data 1. [file elife-91345-fig1-data1.zip › Figure 1-raw images/Figure 1-source data 07-Raw Images_NPC1.tif]

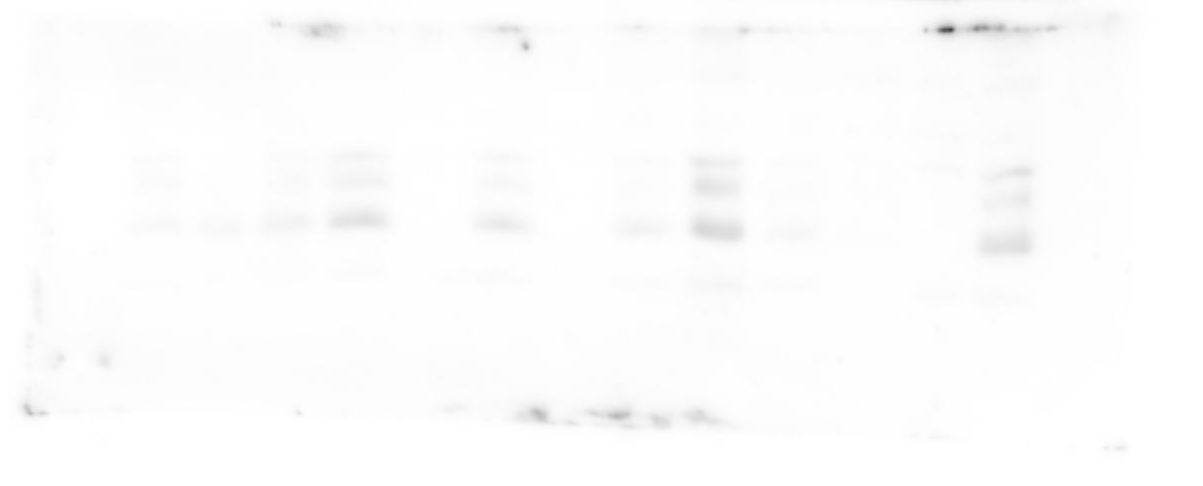

Supplement: Figure 1—source data 1. [file elife-91345-fig1-data1.zip › Figure 1-raw images/Figure 1-source data 09-Raw Images_NPC2.tif]

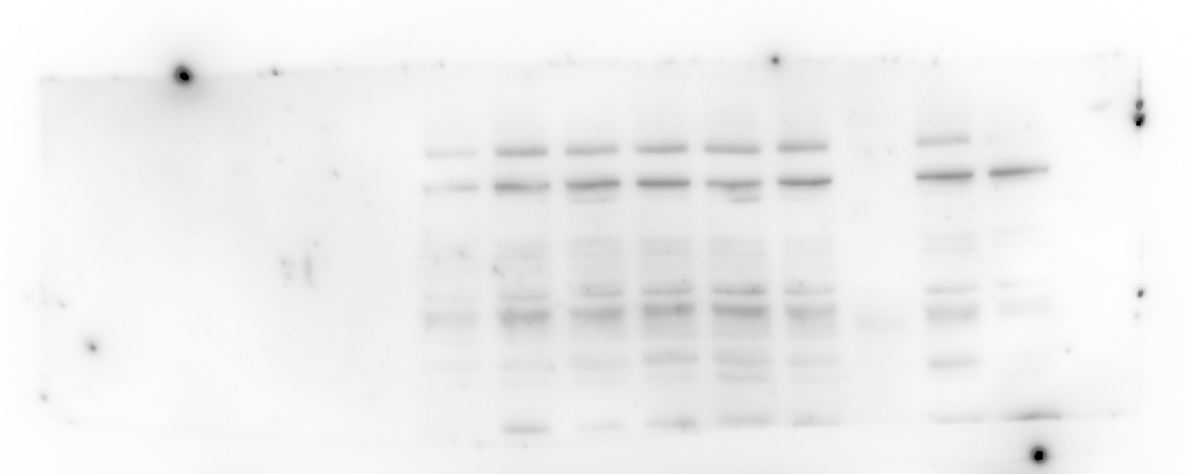

Supplement: Figure 1—source data 1. [file elife-91345-fig1-data1.zip › Figure 1-raw images/Figure 1-source data 16-Raw Images_PITNM1.tif]

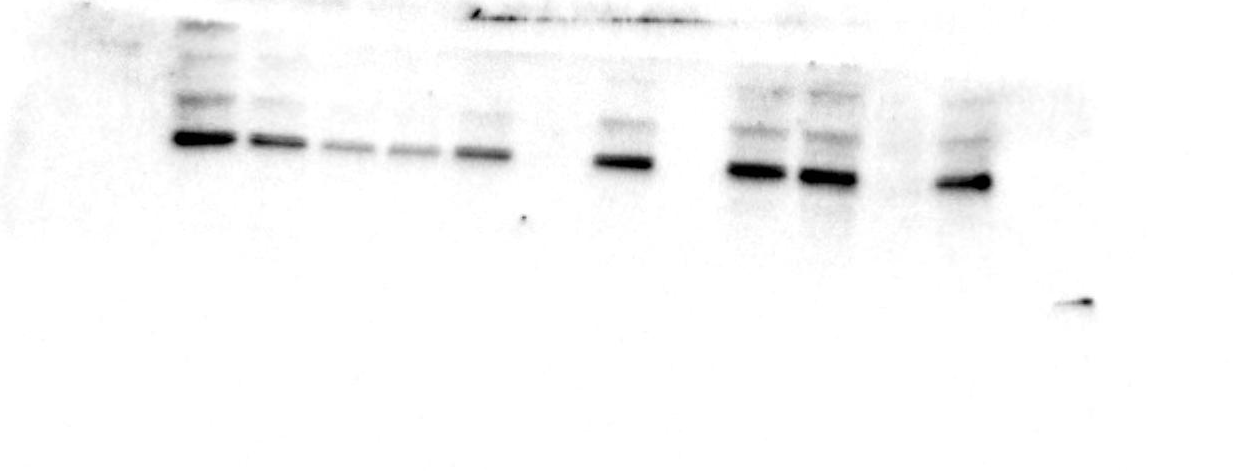

Supplement: Figure 1—source data 1. [file elife-91345-fig1-data1.zip › Figure 1-raw images/Figure 1-source data 13-Raw Images_ORP9.tif]

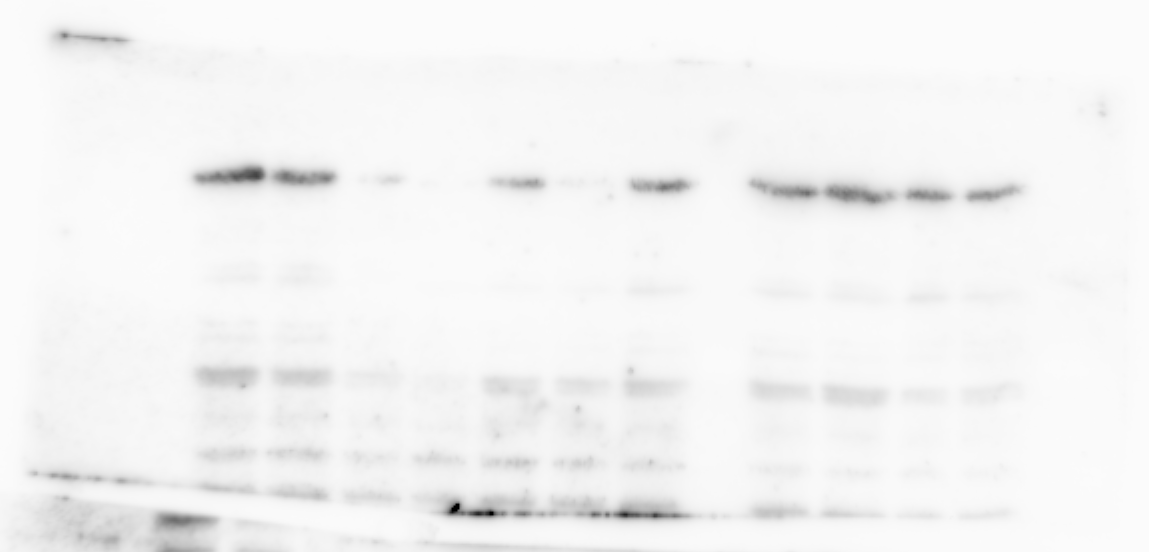

Supplement: Figure 1—source data 1. [file elife-91345-fig1-data1.zip › Figure 1-raw images/Figure 1-source data 05-Raw Images_GLTP.tif]

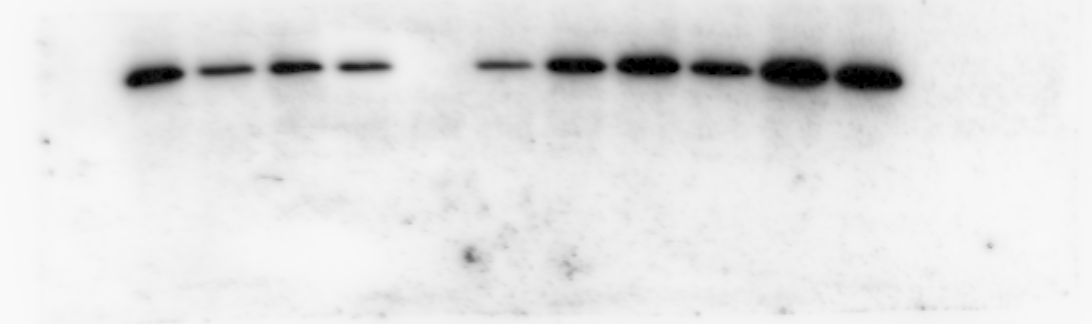

Supplement: Figure 1—source data 1. [file elife-91345-fig1-data1.zip › Figure 1-raw images/Figure 1-source data 18-Raw Images_PITPNB.tif]

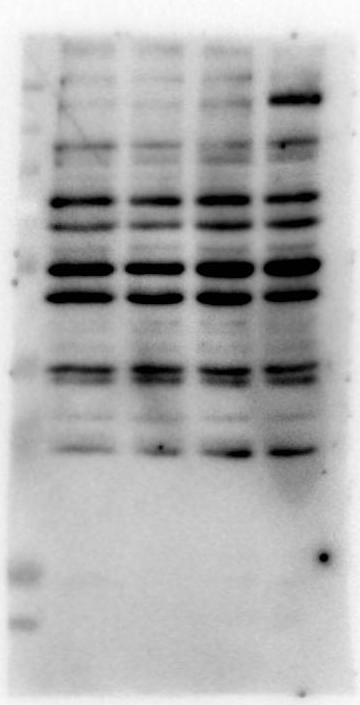

Supplement: Figure 1—source data 1. [file elife-91345-fig1-data1.zip › Figure 1-raw images/Figure 1-source data 27-Raw Images_TEX2.tif]

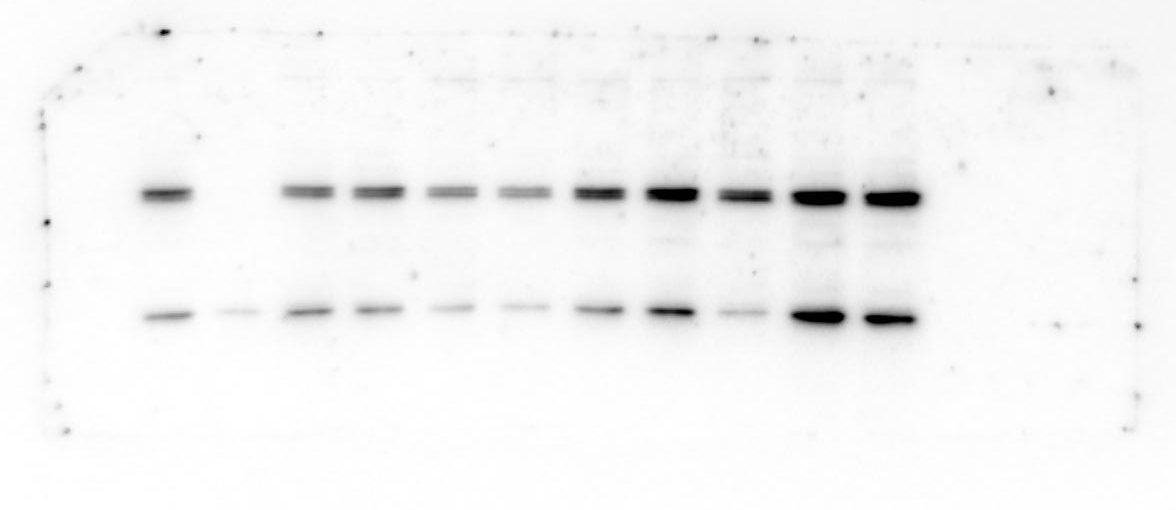

Supplement: Figure 1—source data 1. [file elife-91345-fig1-data1.zip › Figure 1-raw images/Figure 1-source data 15-Raw Images_OSBP.tif]

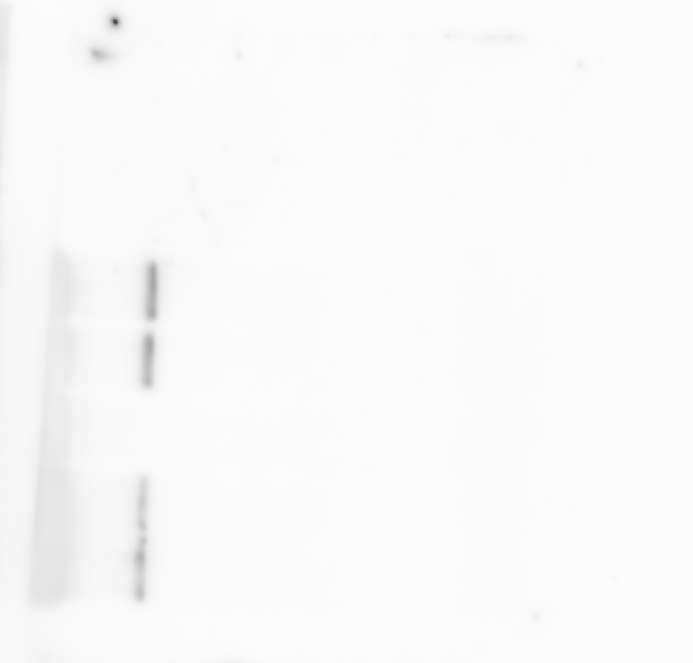

Supplement: Figure 1—source data 1. [file elife-91345-fig1-data1.zip › Figure 1-raw images/Figure 1-source data 23-Raw Images_SEC14L2.tif]

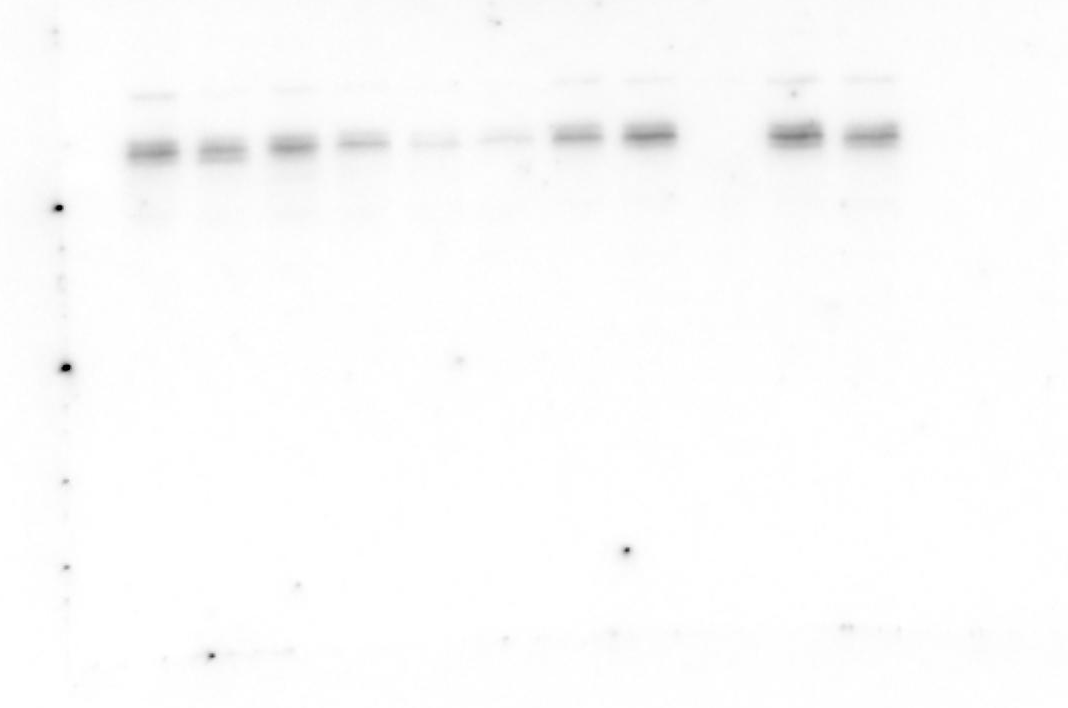

Supplement: Figure 1—source data 1. [file elife-91345-fig1-data1.zip › Figure 1-raw images/Figure 1-source data 03-Raw Images_CERT.tif]

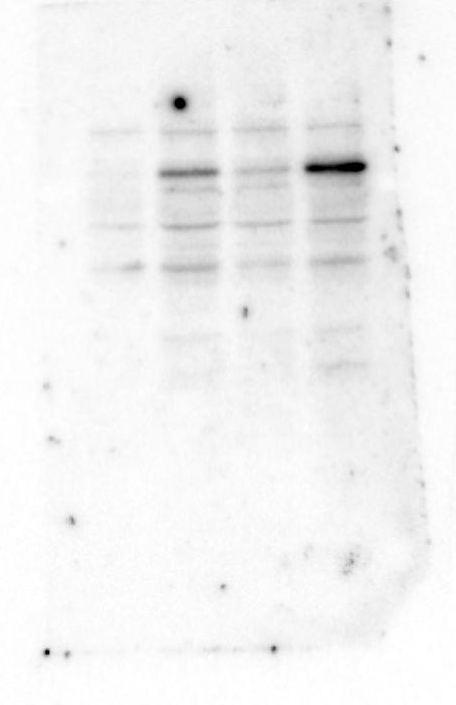

Supplement: Figure 1—source data 1. [file elife-91345-fig1-data1.zip › Figure 1-raw images/Figure 1-source data 22-Raw Images_SEC14L1.tif]

Figure 1

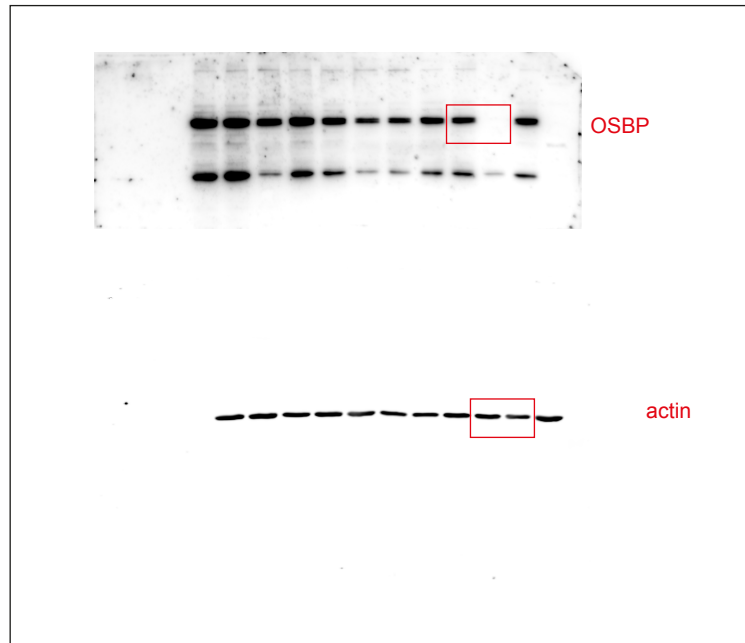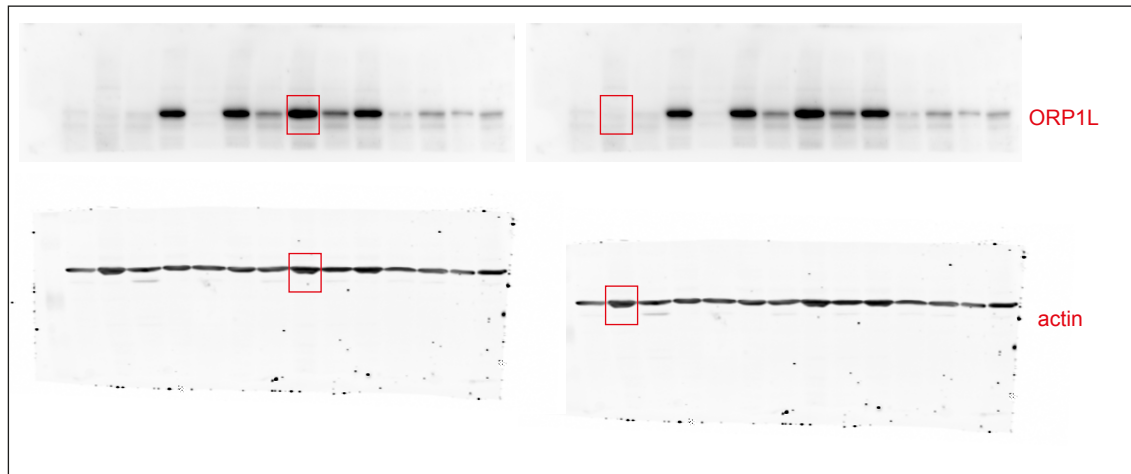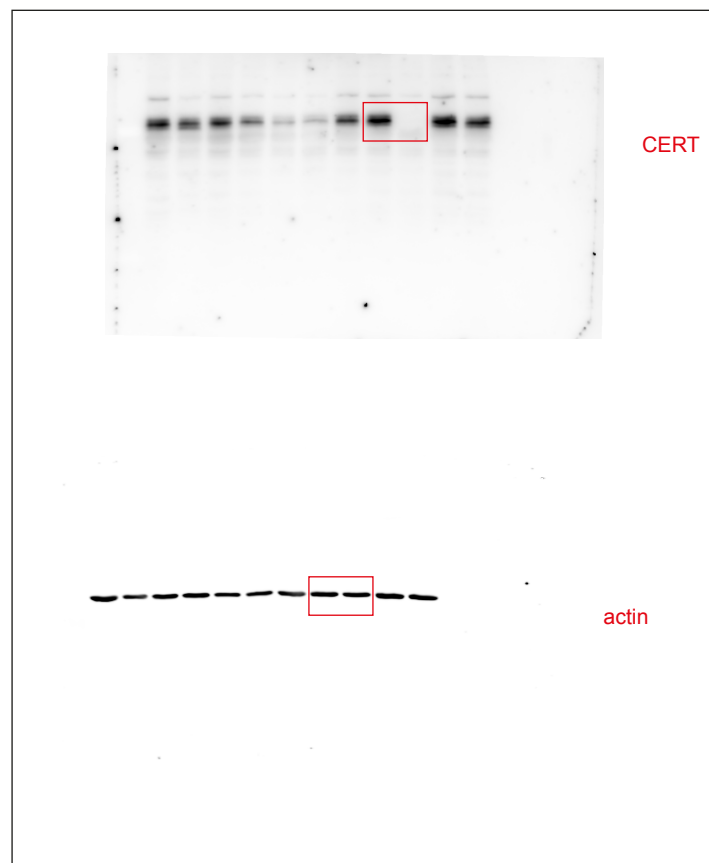

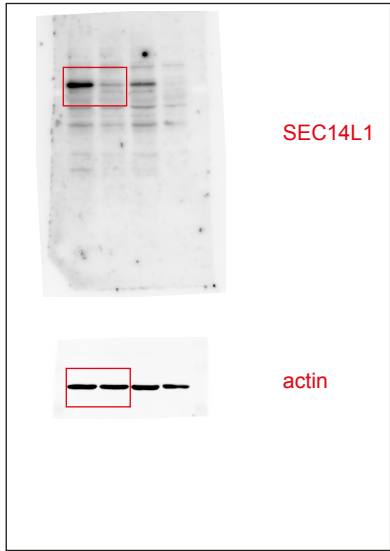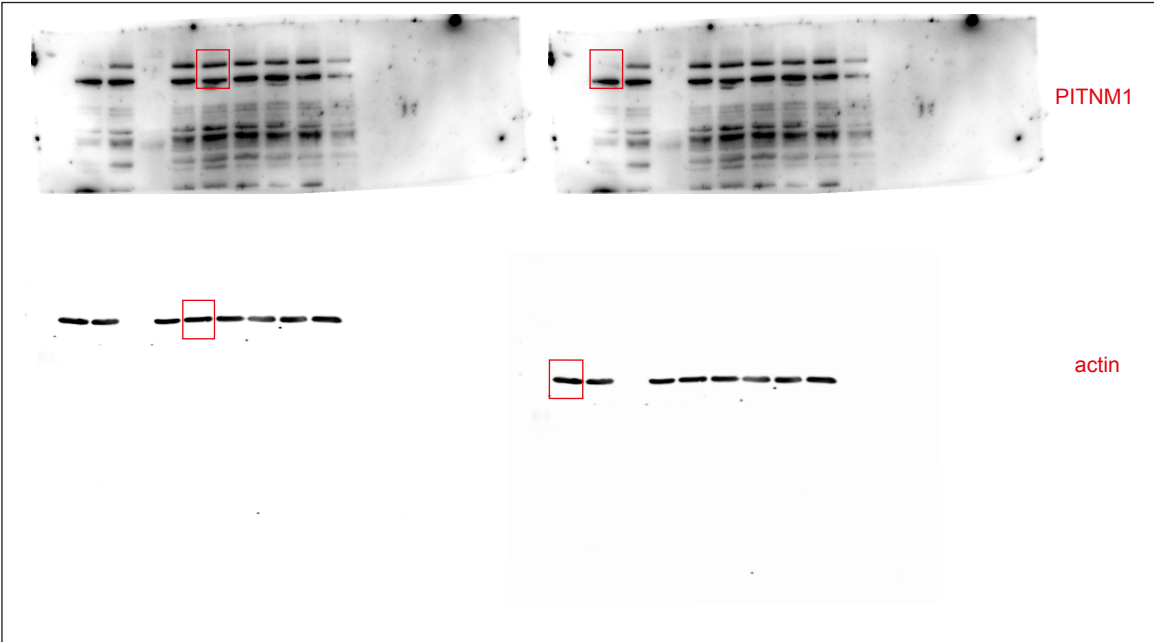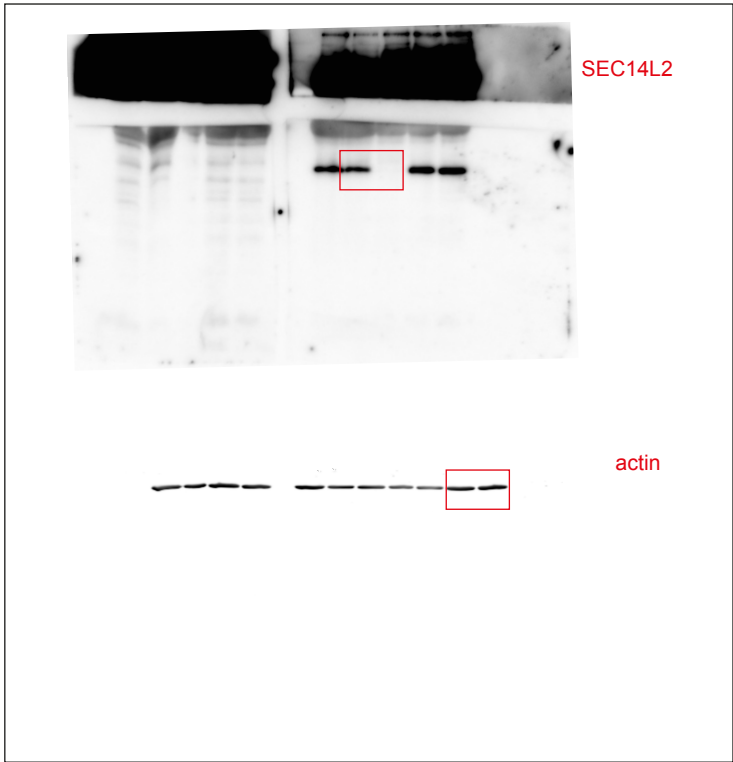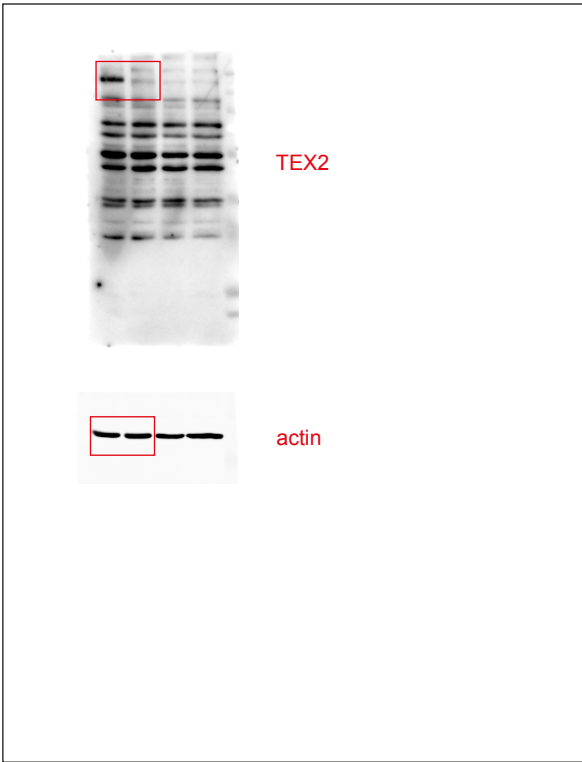

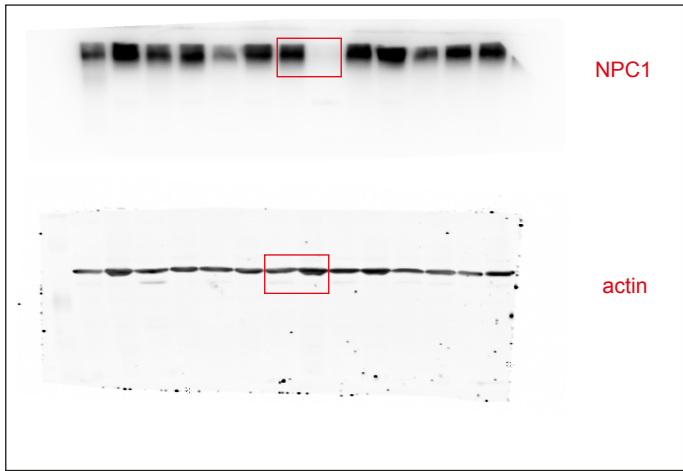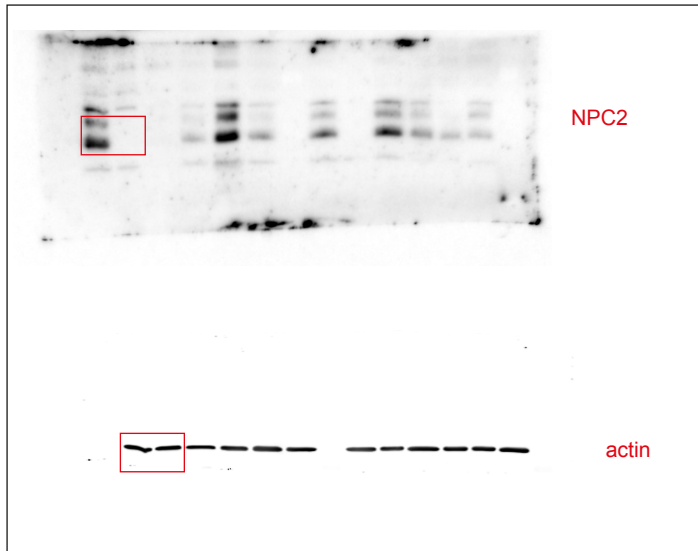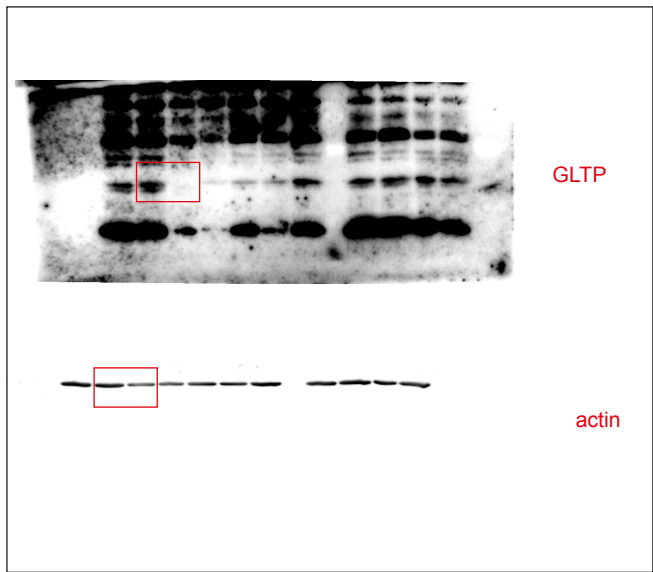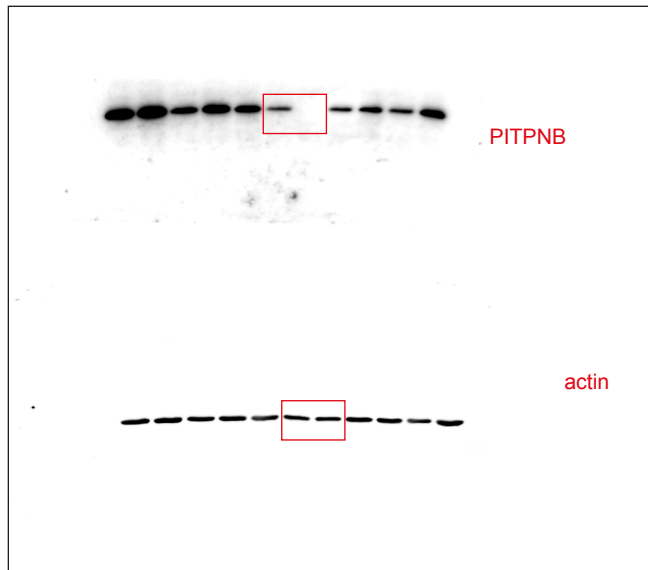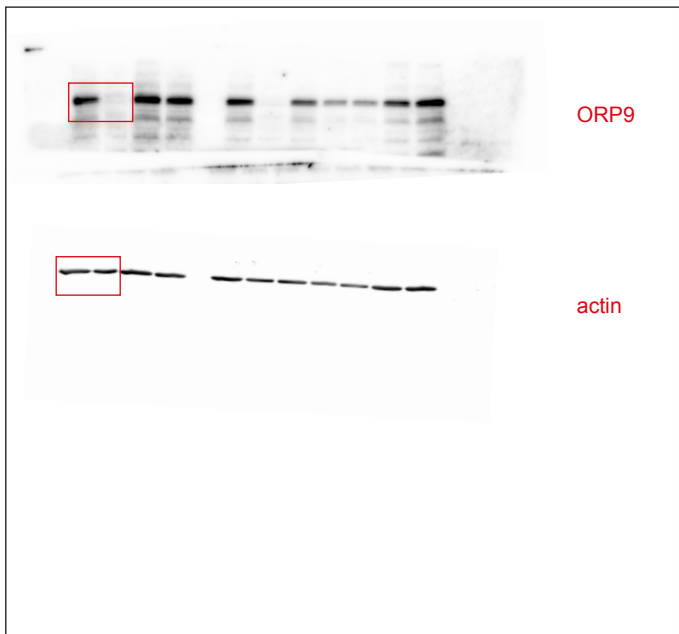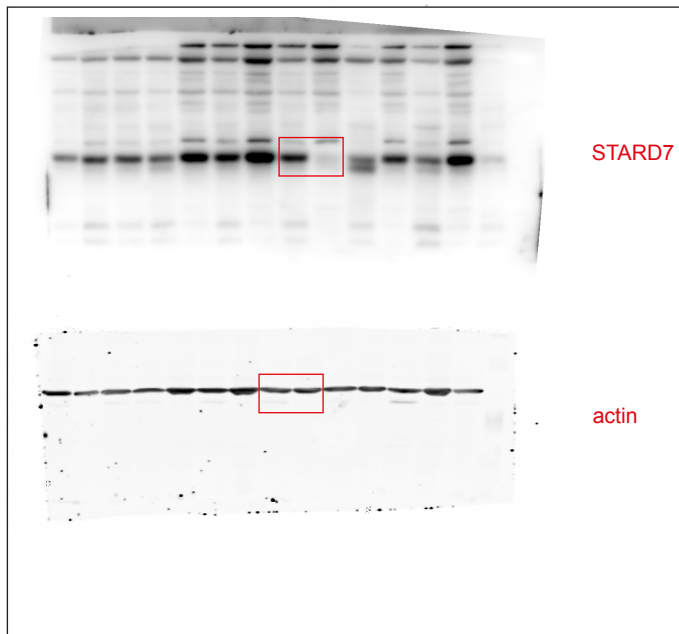

Supplement: Figure 1—source data 2. [file elife-91345-fig1-data2.zip › Figure 1-uncropped_images/Figure 1-source data 01-uncropped_images.pdf]

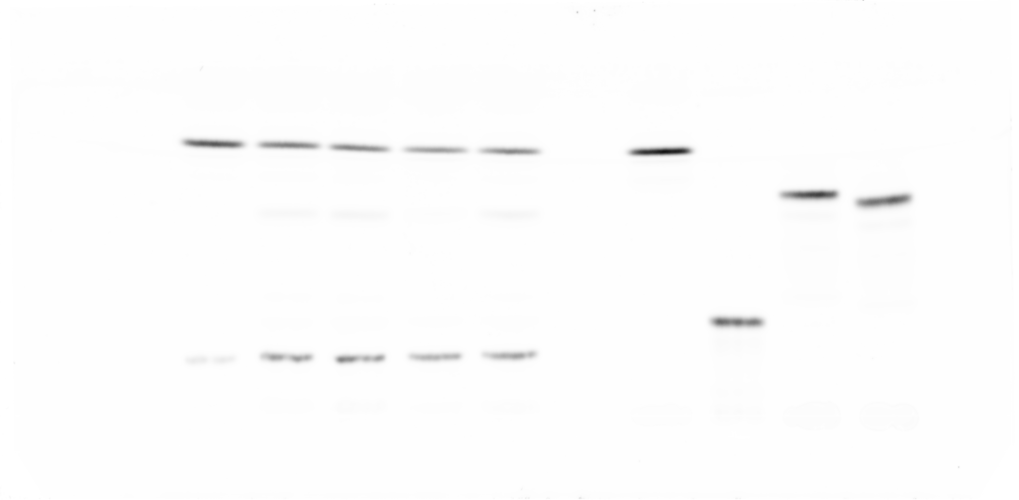

Supplement: Figure 1—figure supplement 1—source data 1. [file elife-91345-fig1-figsupp1-data1.zip › Figure 1-figure supplement 1-raw images/Figure 1-figure supplement 1-source data 2-raw image file.tiff]

Figure1–figure supplement 1

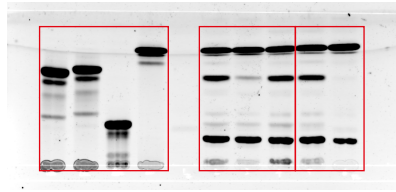

Supplement: Figure 1—figure supplement 1—source data 2. [file elife-91345-fig1-figsupp1-data2.zip › Figure 1-figure supplement 1-uncropped_images/Figure 1-figure supplement 1-source data 1-uncropped_images.pdf]

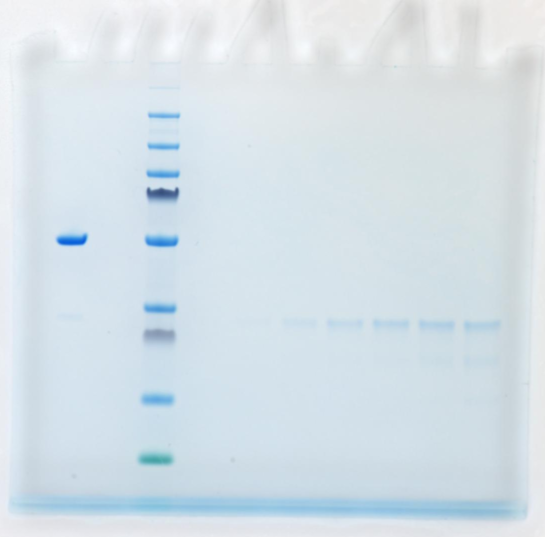

Supplement: Figure 2—figure supplement 1—source data 1. [file elife-91345-fig2-figsupp1-data1.zip › Figure 2-figure supplement 1-raw images/Figure 2-figure supplement 1-source data 2-raw image.jpeg]

Figure 2-figure supplement 1

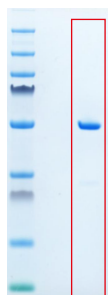

Supplement: Figure 2—figure supplement 1—source data 2. [file elife-91345-fig2-figsupp1-data2.zip › Figure 2-figure supplement 1-uncropped_images/Figure 2-figure supplement 1-source data 1-uncropped_images.pdf]

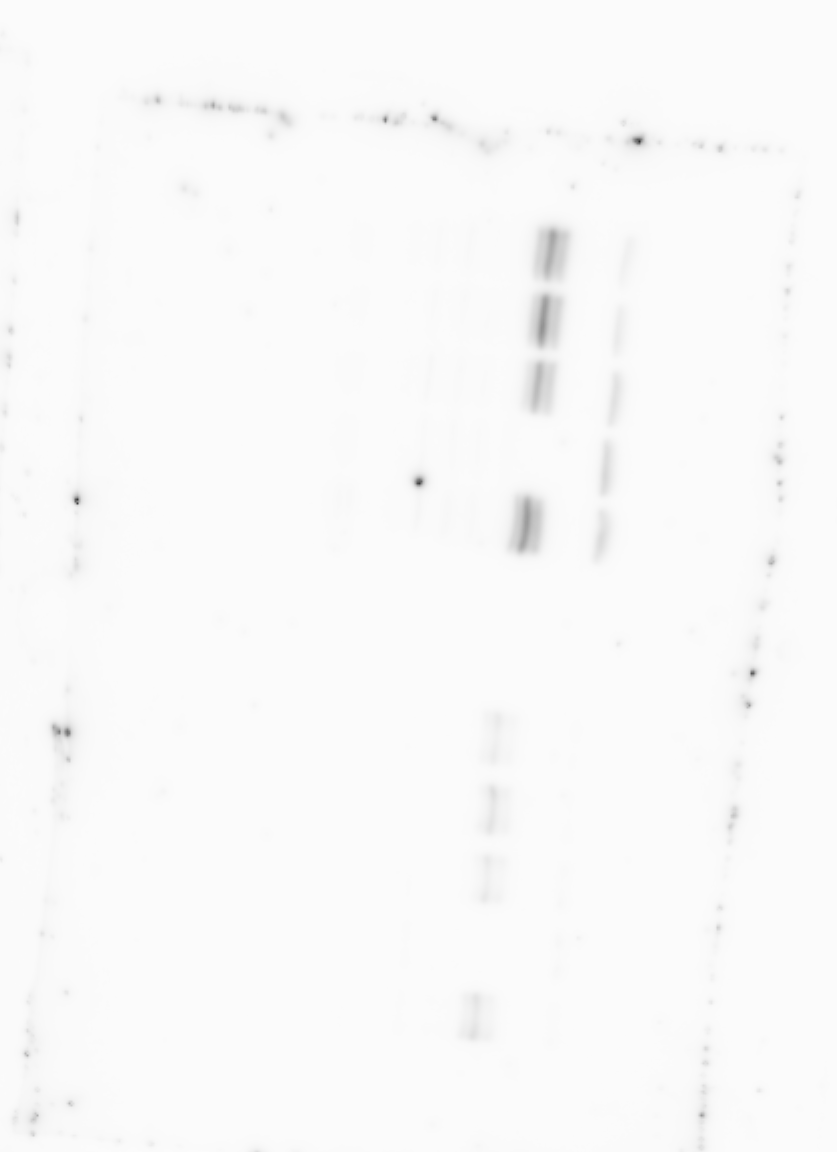

Supplement: Figure 3—source data 1. [file elife-91345-fig3-data1.zip › Figure 3-raw images/Figure 3-source data 2-Raw Images.tiff]

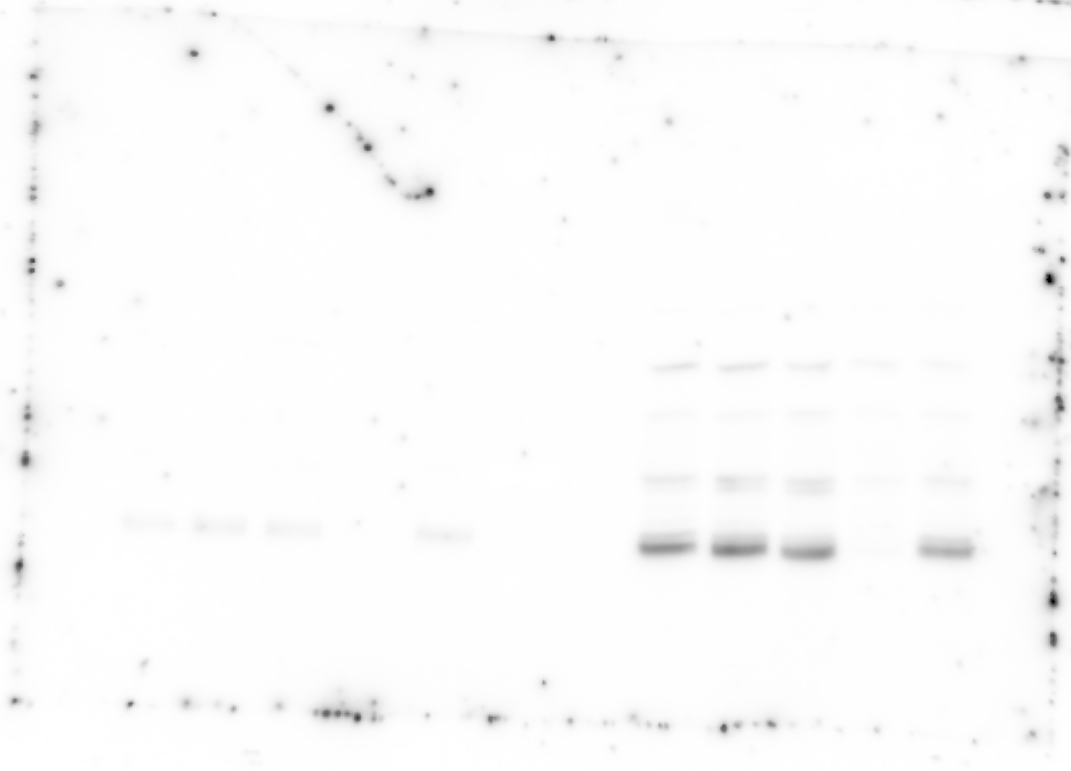

Supplement: Figure 3—source data 1. [file elife-91345-fig3-data1.zip › Figure 3-raw images/Figure 3-source data 5-Raw Images.tiff]

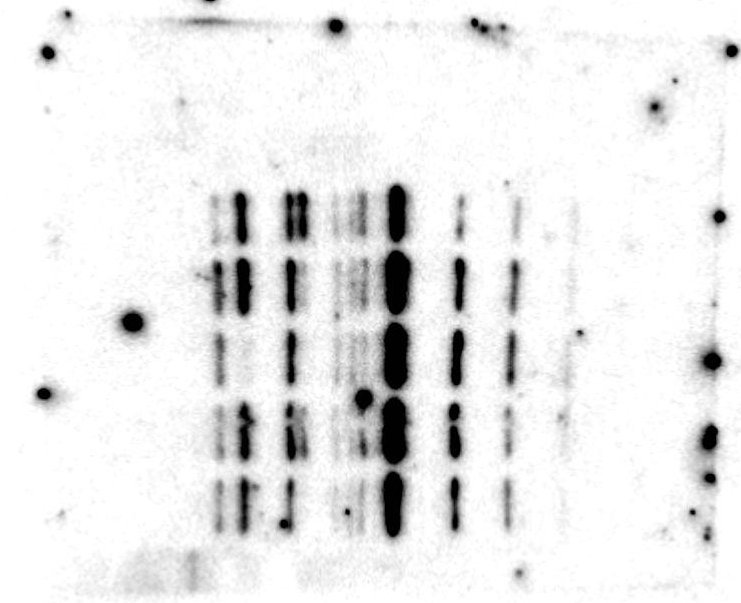

Supplement: Figure 3—source data 1. [file elife-91345-fig3-data1.zip › Figure 3-raw images/Figure 3-source data 4-Raw Images.tiff]

Figure 3

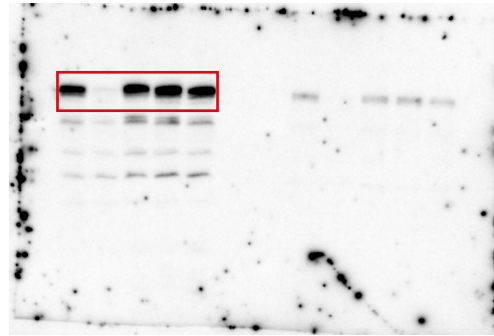

ORP9

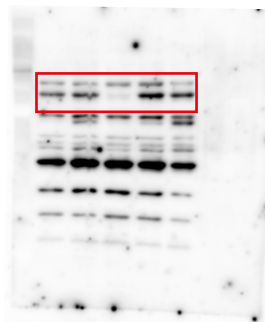

ORP11

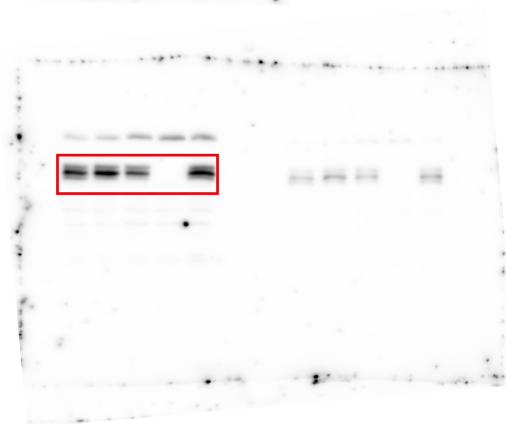

CERT

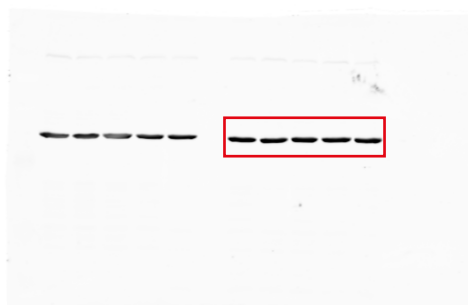

actin

Supplement: Figure 3—source data 2. [file elife-91345-fig3-data2.zip › Figure 3-uncropped_images/Figure 3-source data 1-uncropped_images.pdf]

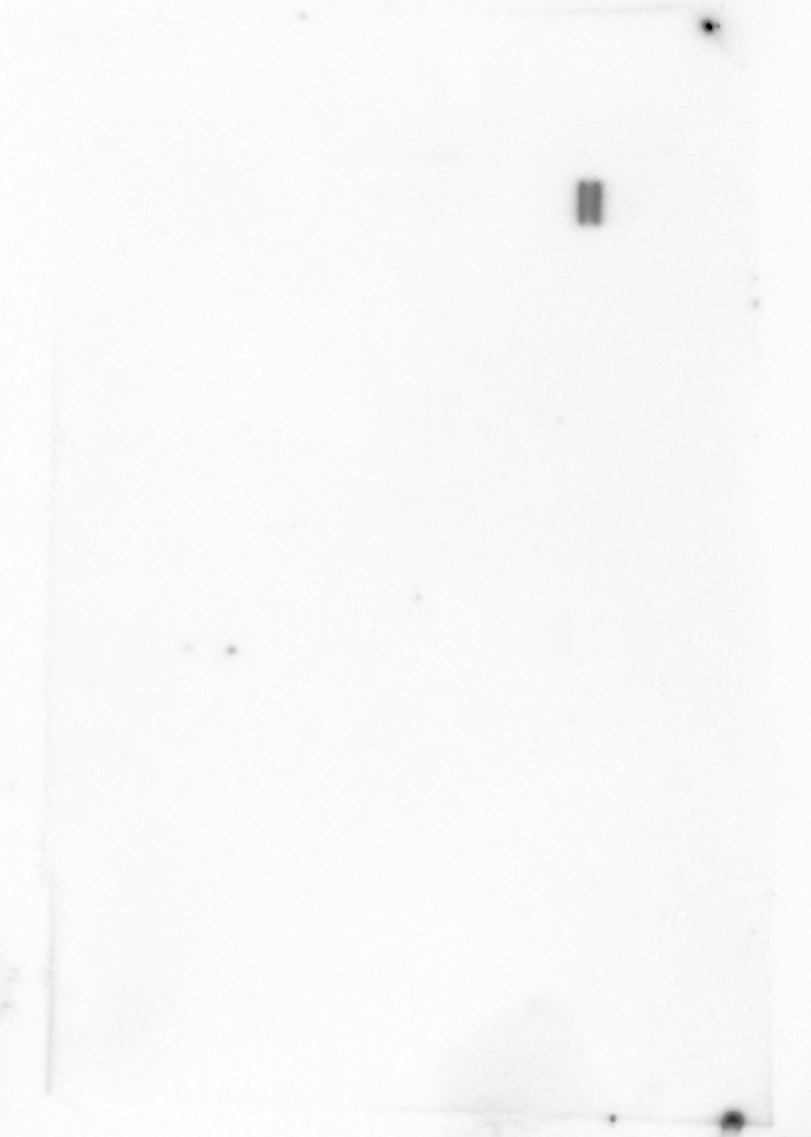

Supplement: Figure 4—source data 1. [file elife-91345-fig4-data1.zip › Figure 4-raw images/Figure 4-source data 5-Raw Images.tif]

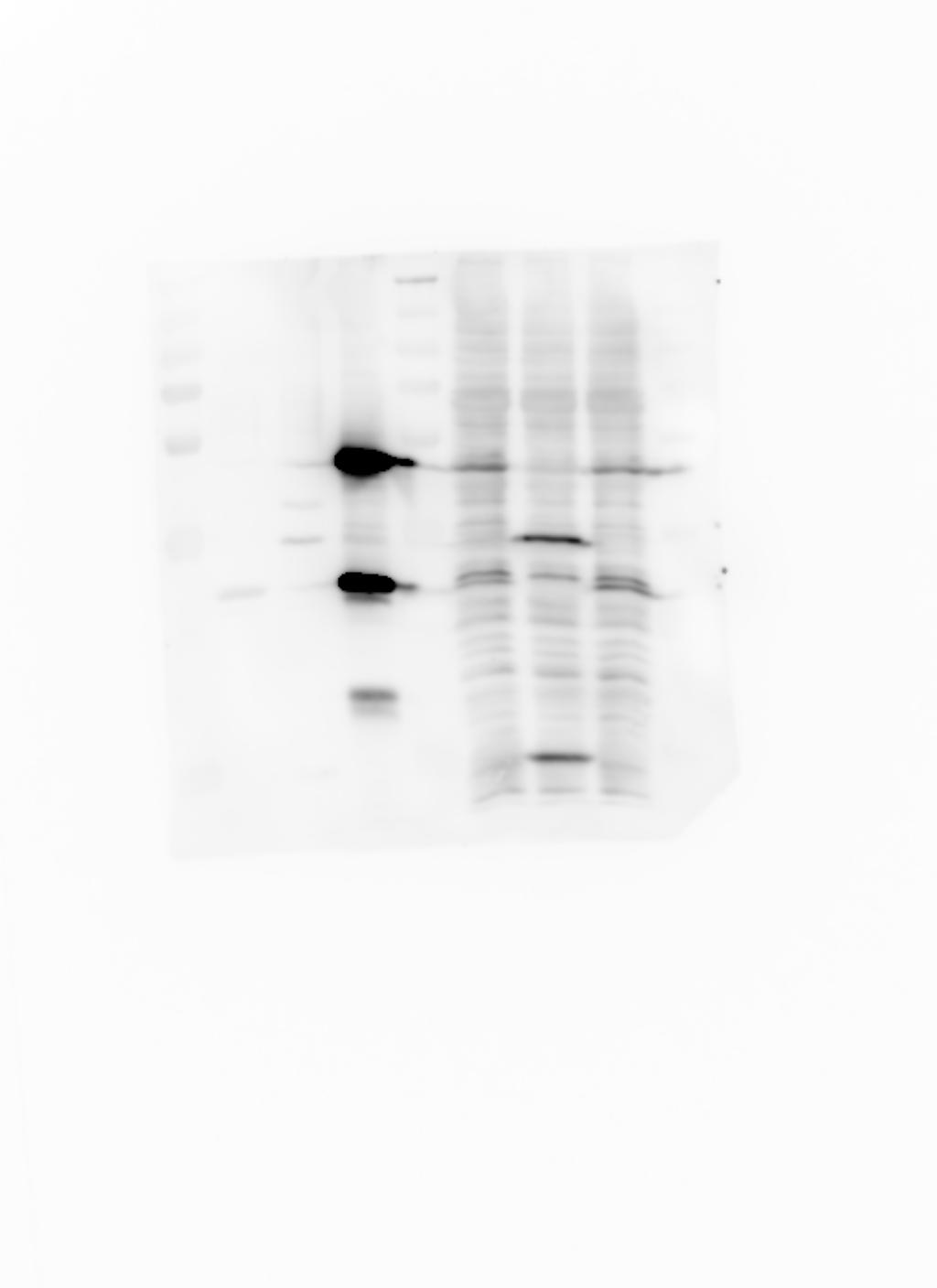

Supplement: Figure 4—source data 1. [file elife-91345-fig4-data1.zip › Figure 4-raw images/Figure 4-source data 9-Raw Images.jpeg]

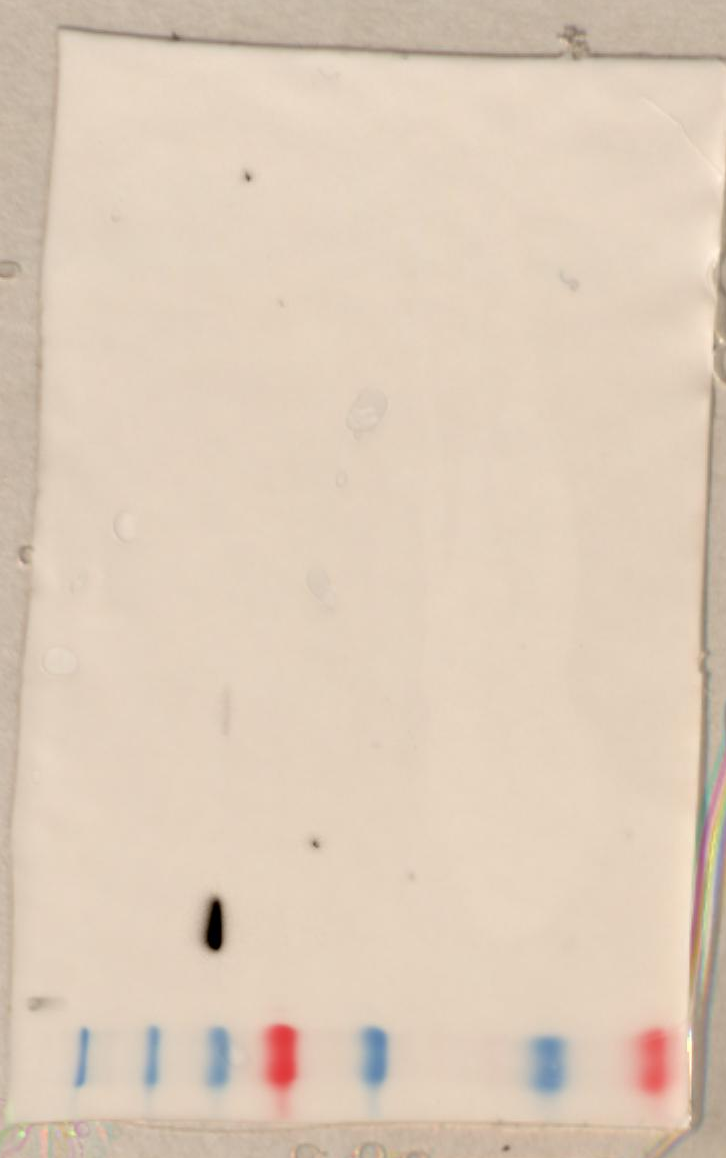

Supplement: Figure 4—source data 1. [file elife-91345-fig4-data1.zip › Figure 4-raw images/Figure 4-source data 2-Raw Images.tif]

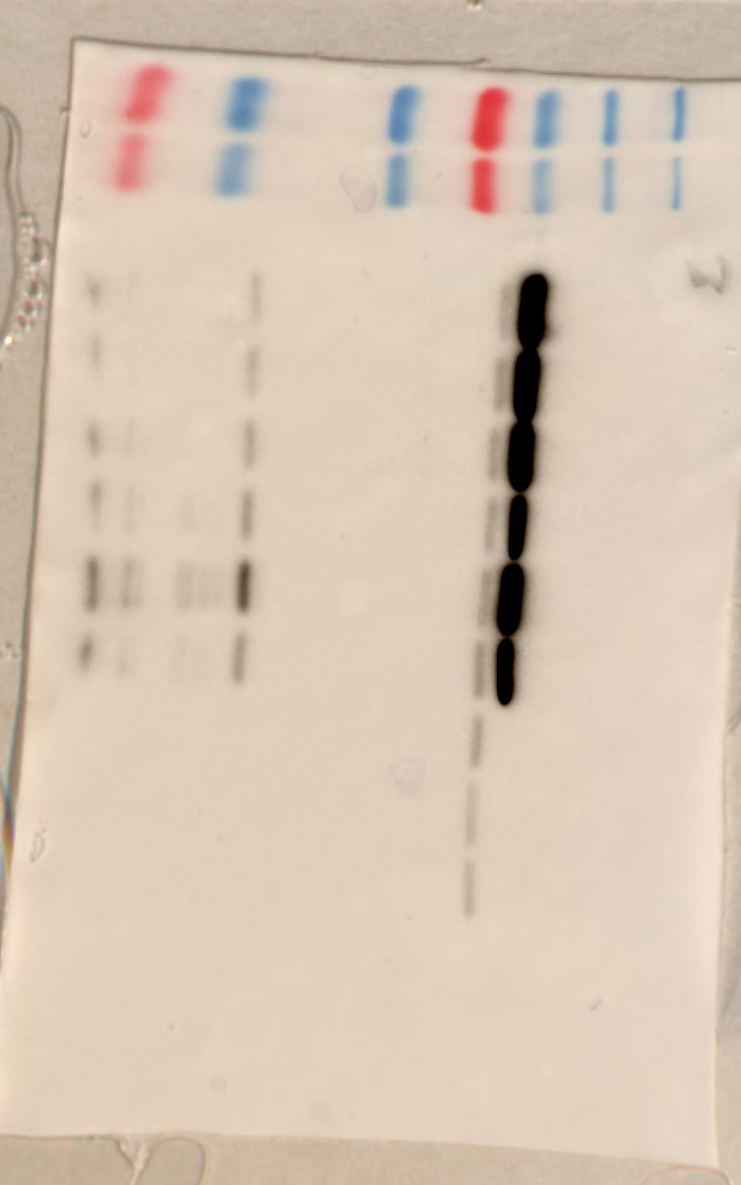

Supplement: Figure 4—source data 1. [file elife-91345-fig4-data1.zip › Figure 4-raw images/Figure 4-source data 3-Raw Images.tif]

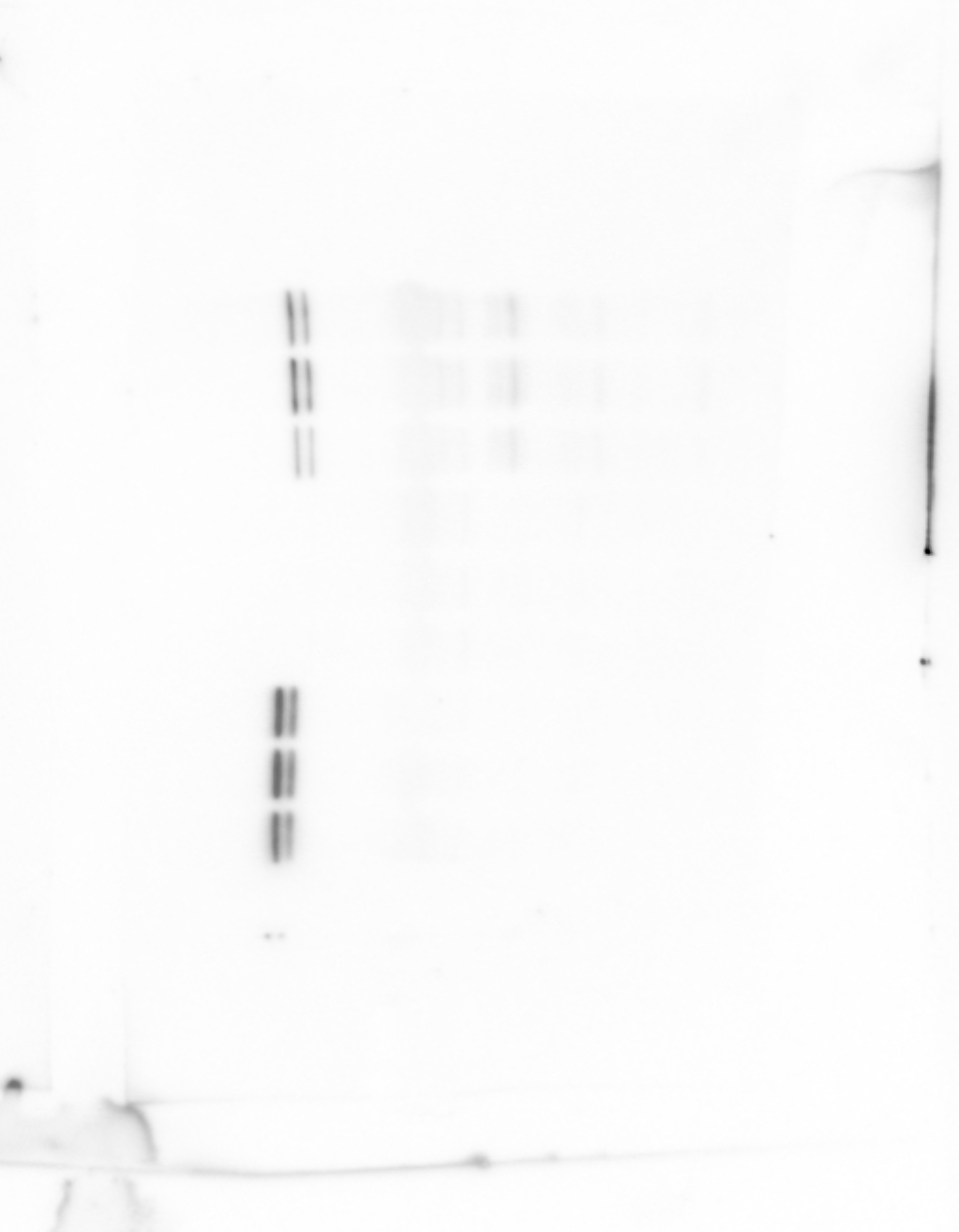

Supplement: Figure 4—source data 1. [file elife-91345-fig4-data1.zip › Figure 4-raw images/Figure 4-source data 4-Raw Images.tif]

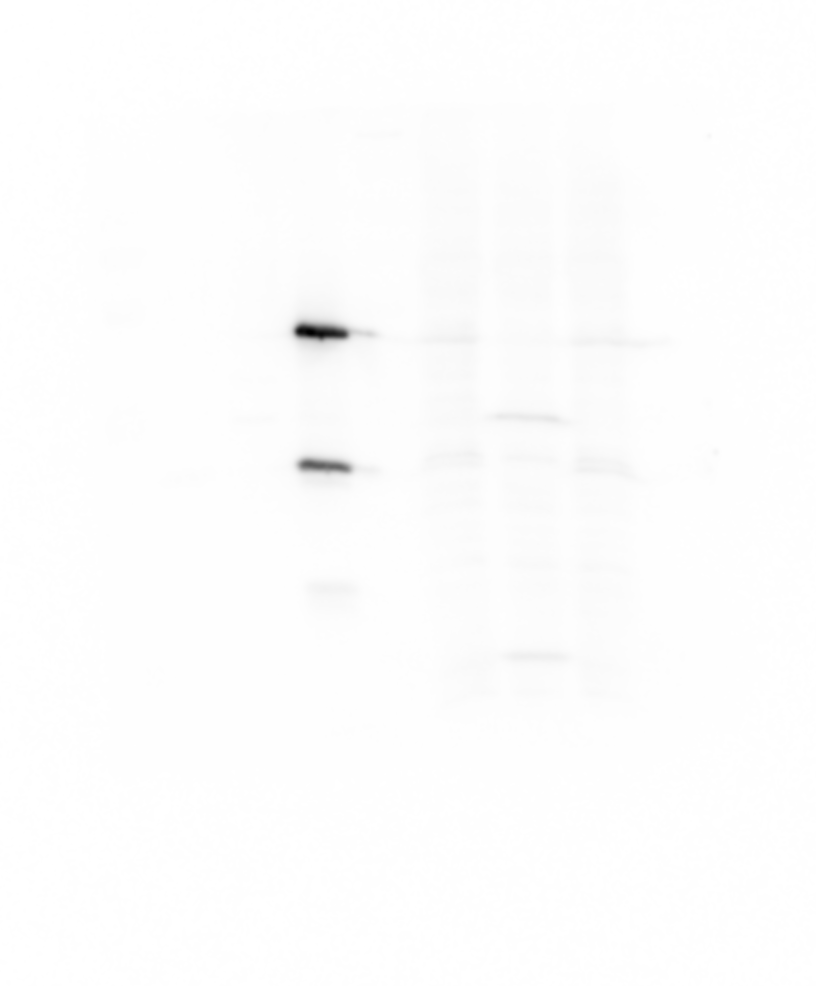

Supplement: Figure 4—source data 1. [file elife-91345-fig4-data1.zip › Figure 4-raw images/Figure 4-source data 8-Raw Images.tif]

Figure 4

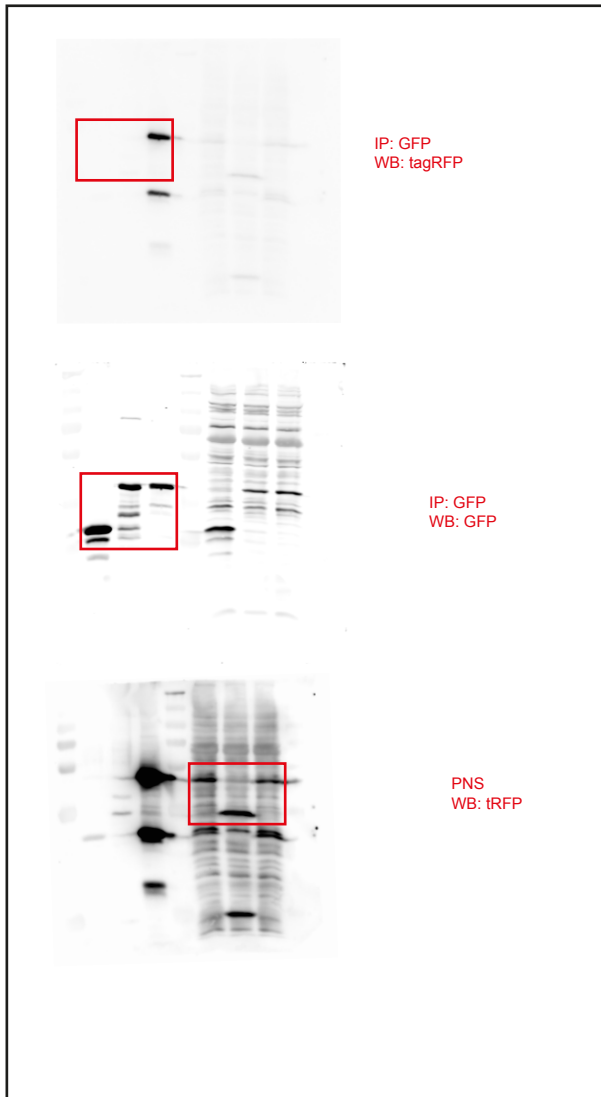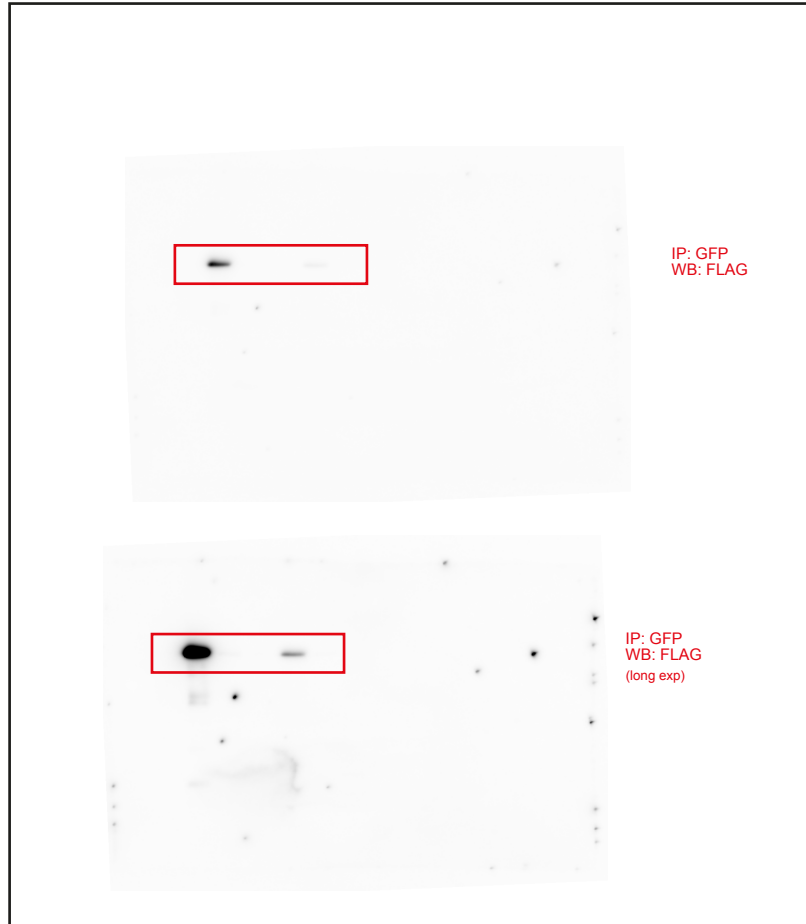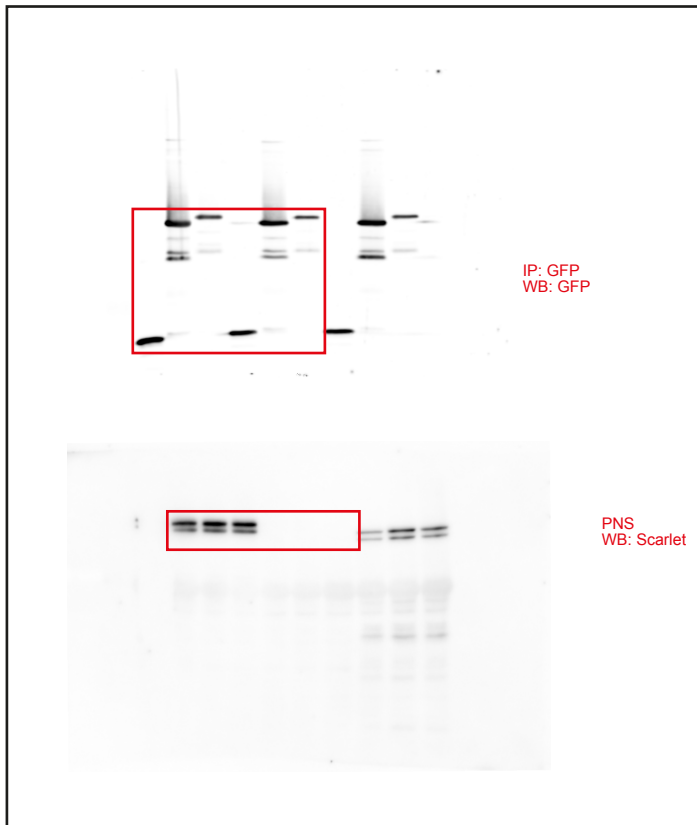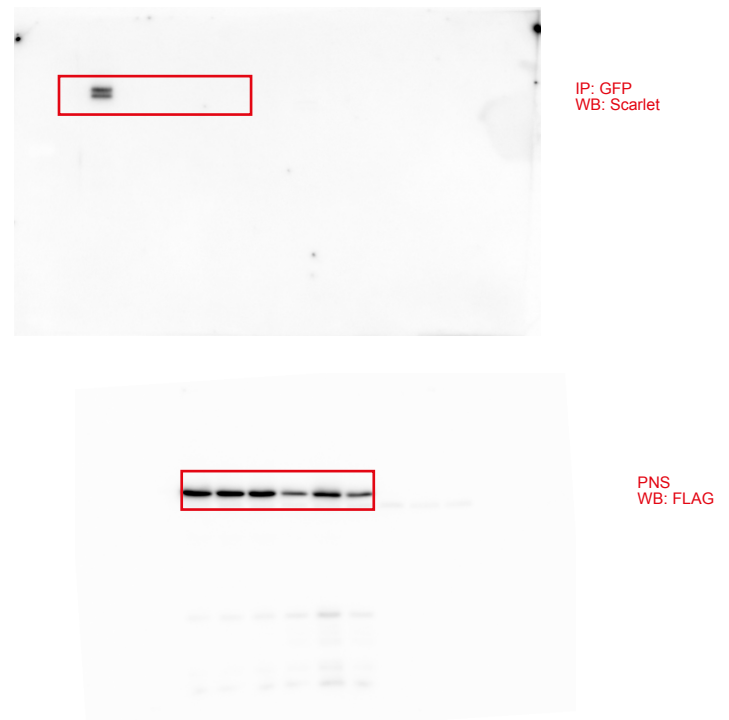

Supplement: Figure 4—source data 2. [file elife-91345-fig4-data2.zip › Figure 4-uncropped images/Figure 4-source data 1-uncropped_images.pdf]

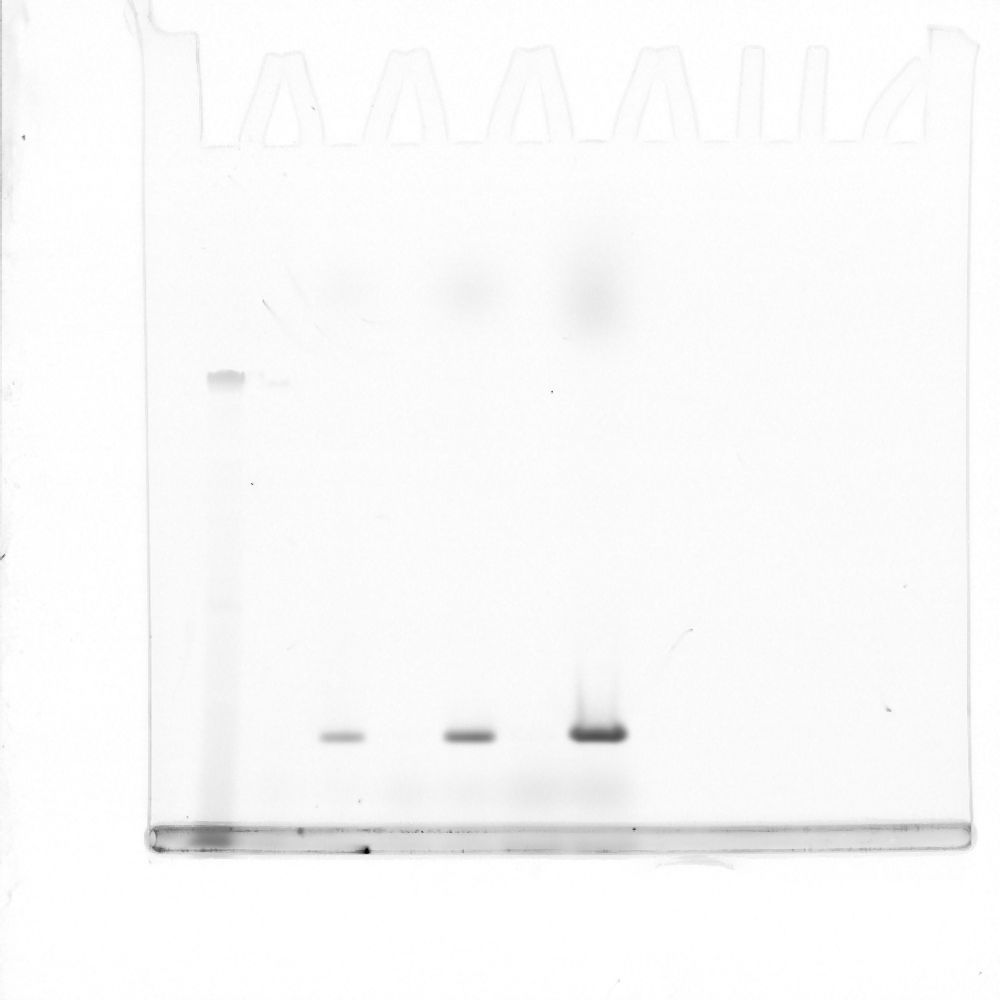

Supplement: Figure 6—figure supplement 1—source data 1. [file elife-91345-fig6-figsupp1-data1.zip › Figure 6-figure supplement 1-raw images/Figure 6-figure supplement 1-source data 4-Raw Images.gel]

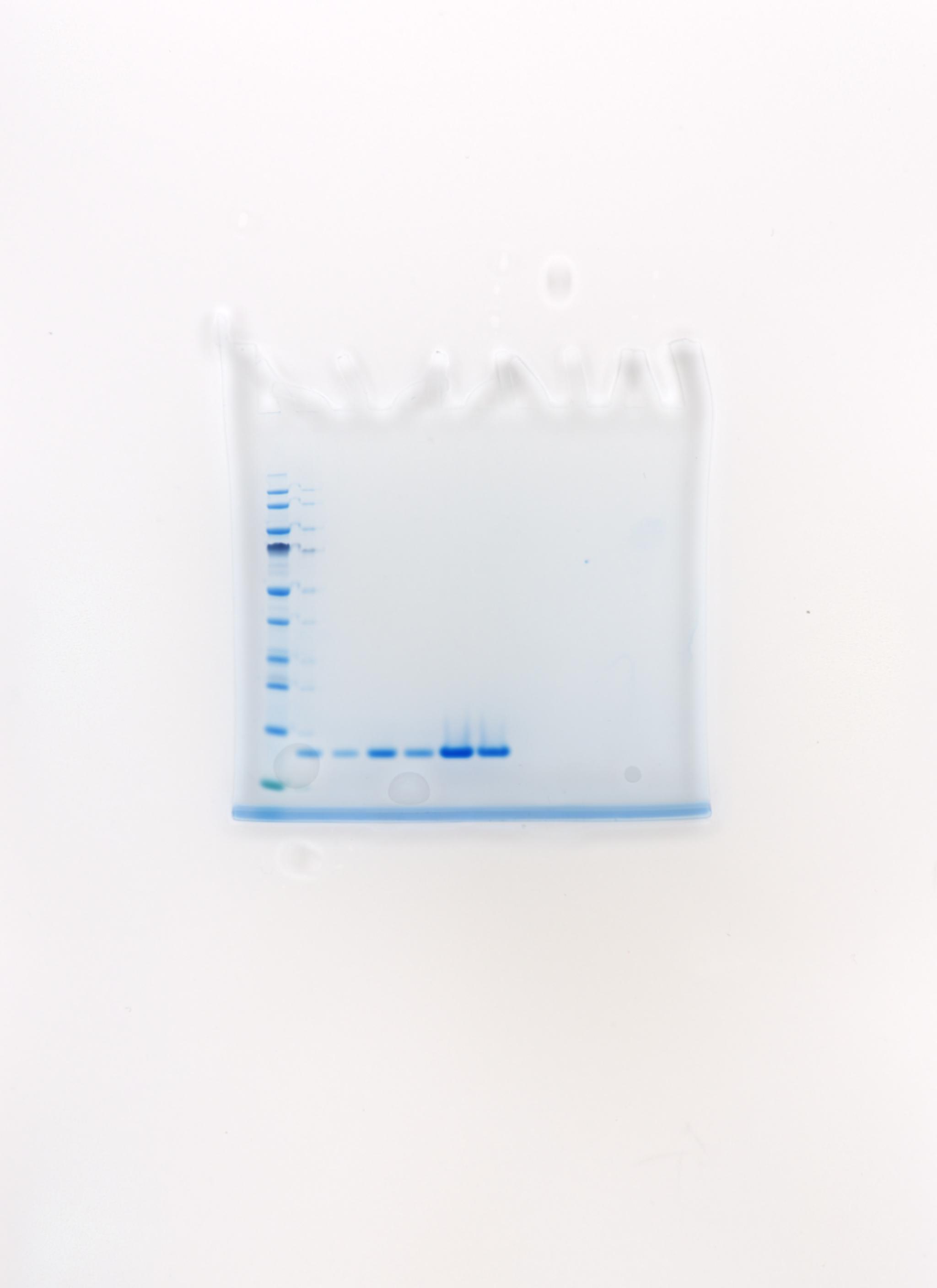

Supplement: Figure 6—figure supplement 1—source data 1. [file elife-91345-fig6-figsupp1-data1.zip › Figure 6-figure supplement 1-raw images/Figure 6-figure supplement 1-source data 3-Raw Images.jpg]

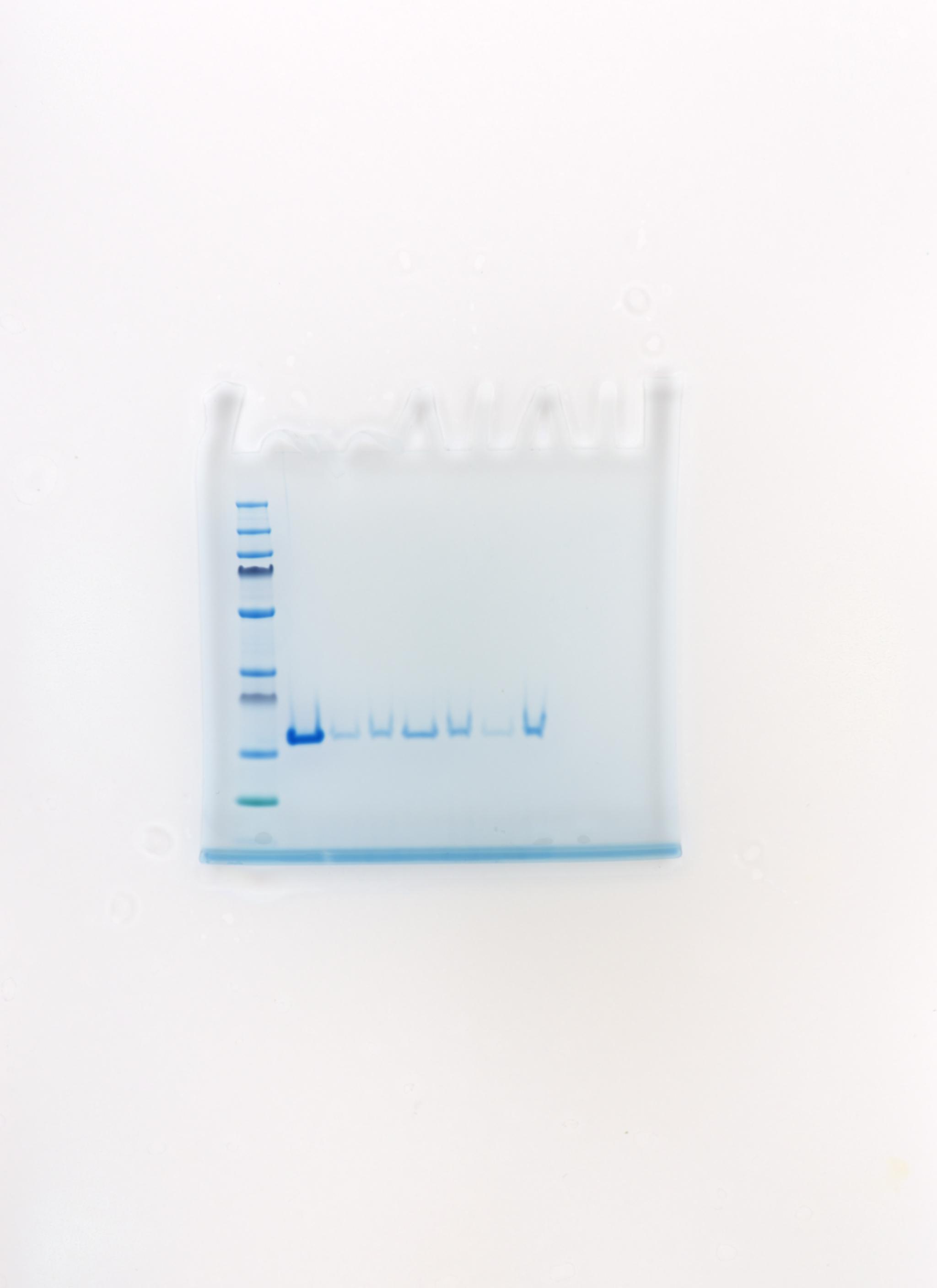

Supplement: Figure 6—figure supplement 1—source data 1. [file elife-91345-fig6-figsupp1-data1.zip › Figure 6-figure supplement 1-raw images/Figure 6-figure supplement 1-source data 6-Raw Images.jpeg]

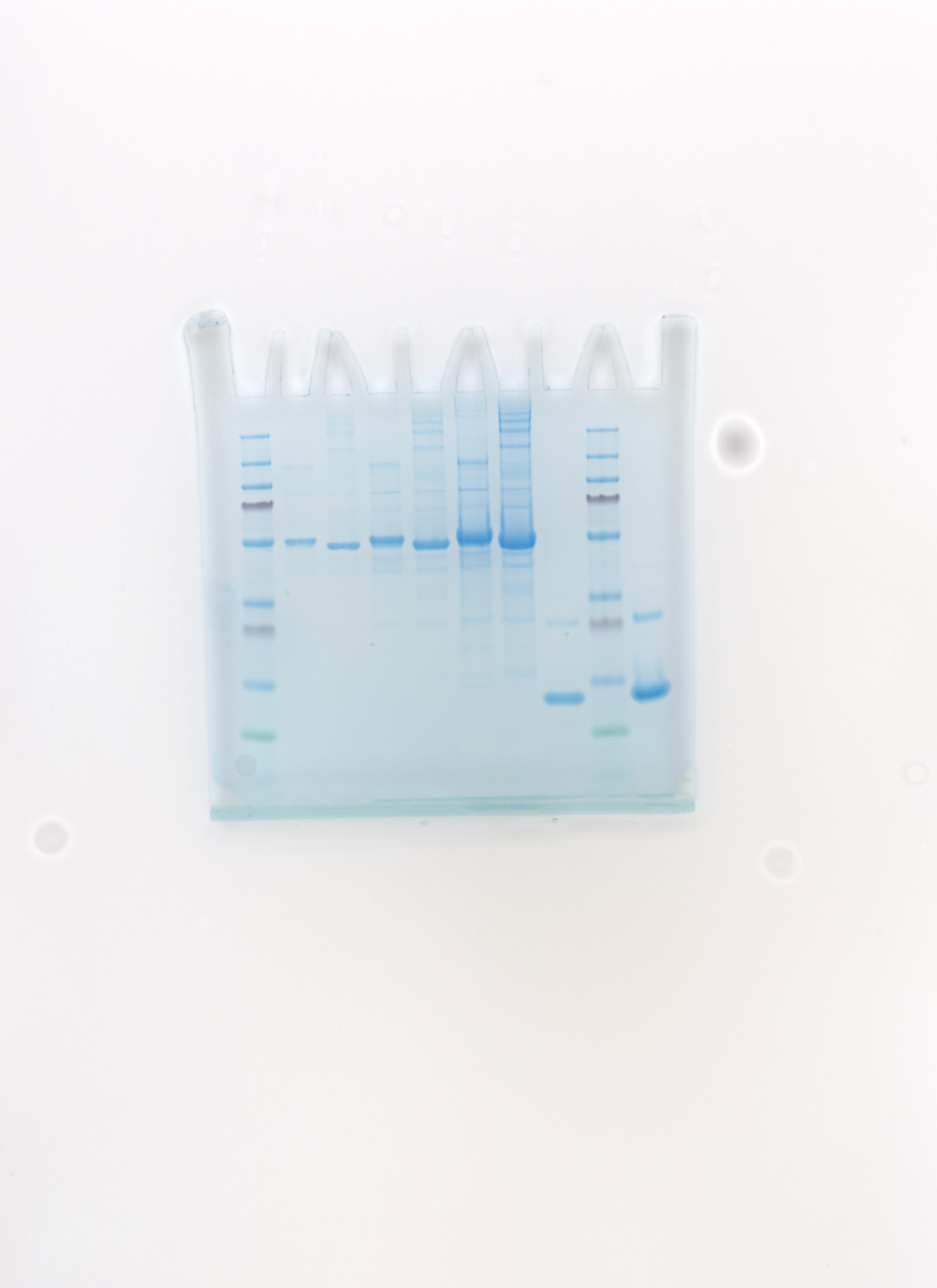

Supplement: Figure 6—figure supplement 1—source data 1. [file elife-91345-fig6-figsupp1-data1.zip › Figure 6-figure supplement 1-raw images/Figure 6-figure supplement 1-source data 2-Raw Images.jpg]

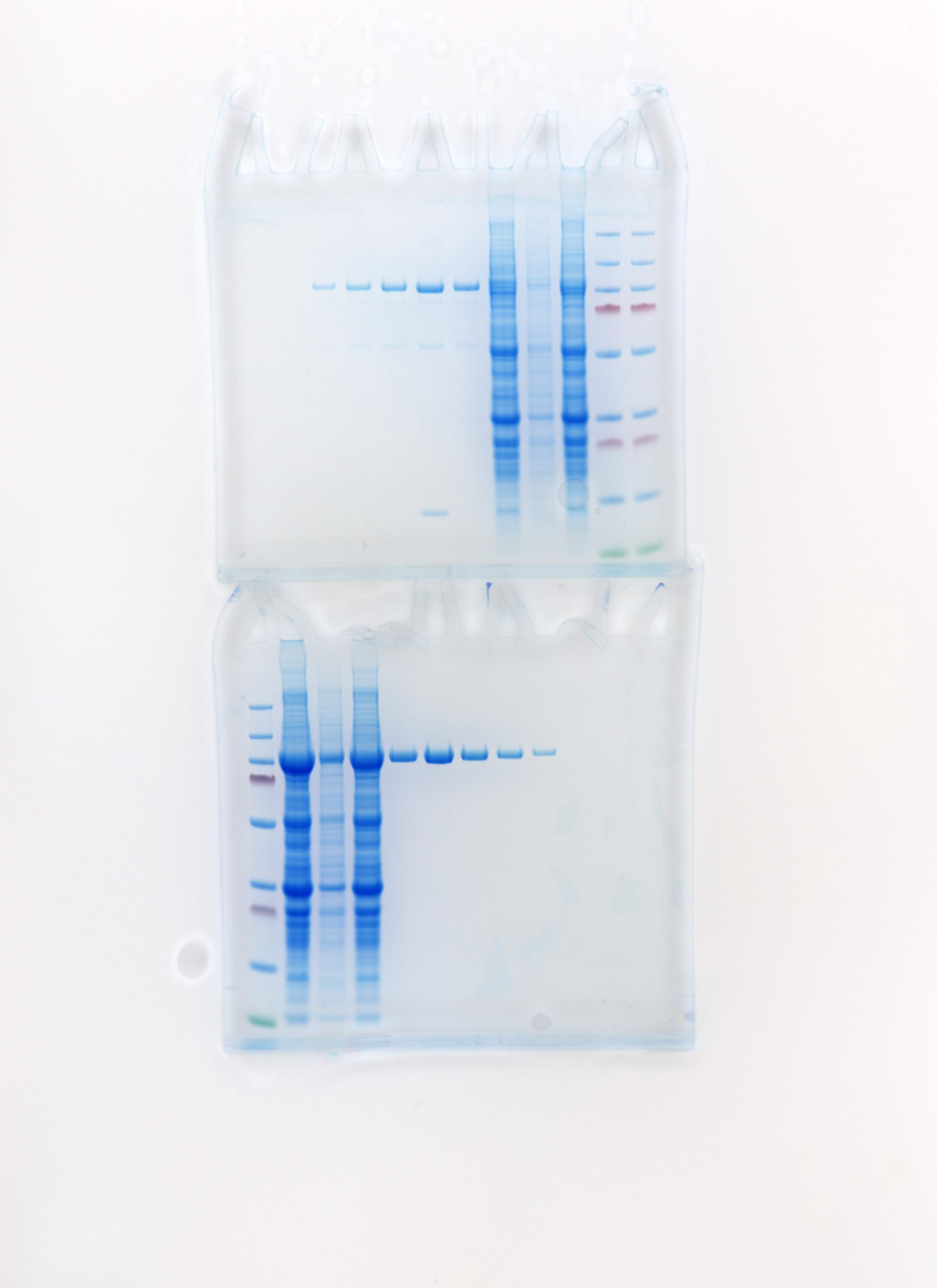

Supplement: Figure 6—figure supplement 1—source data 1. [file elife-91345-fig6-figsupp1-data1.zip › Figure 6-figure supplement 1-raw images/Figure 6-figure supplement 1-source data 5-Raw Images.jpg]

Figure 6-figure supplement 1

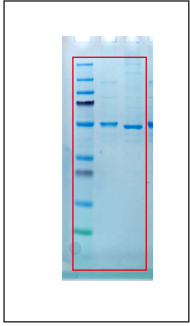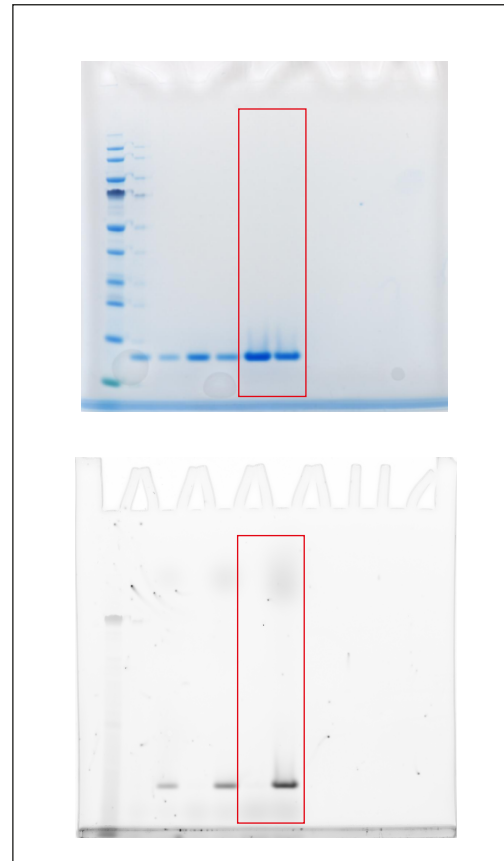

Strep-ORP9

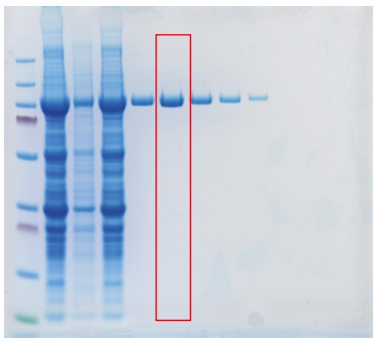

Strep-ORP11

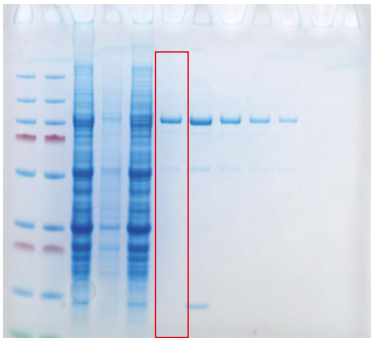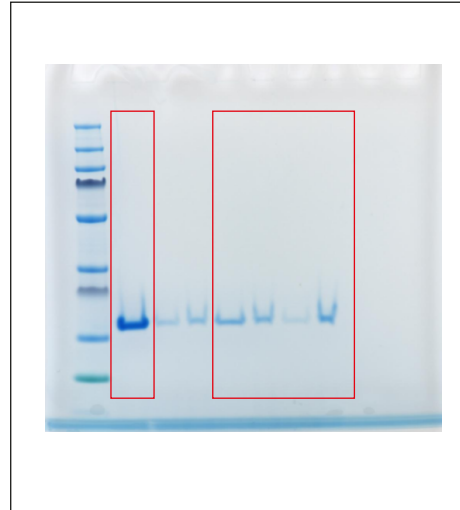

Supplement: Figure 6—figure supplement 1—source data 2. [file elife-91345-fig6-figsupp1-data2.zip › Figure 6-figure supplement 1-uncropped images/Figure 6-figure supplement 1-source data 1-uncropped_images.pdf]

Figure 7

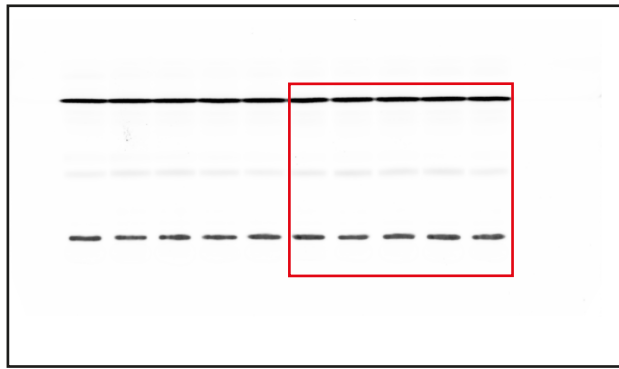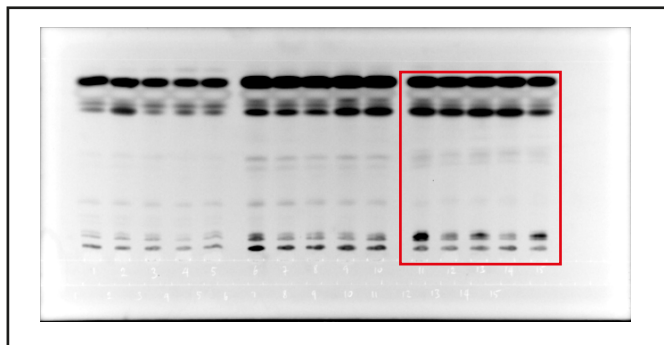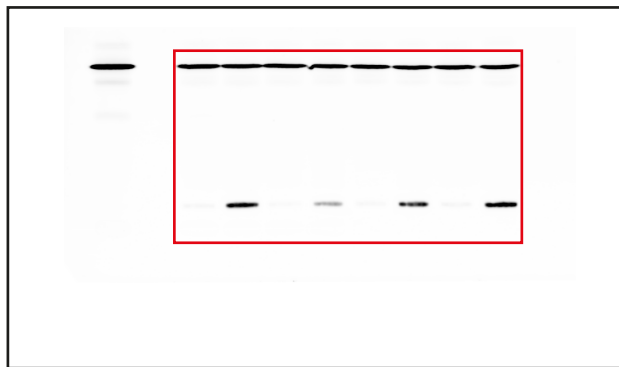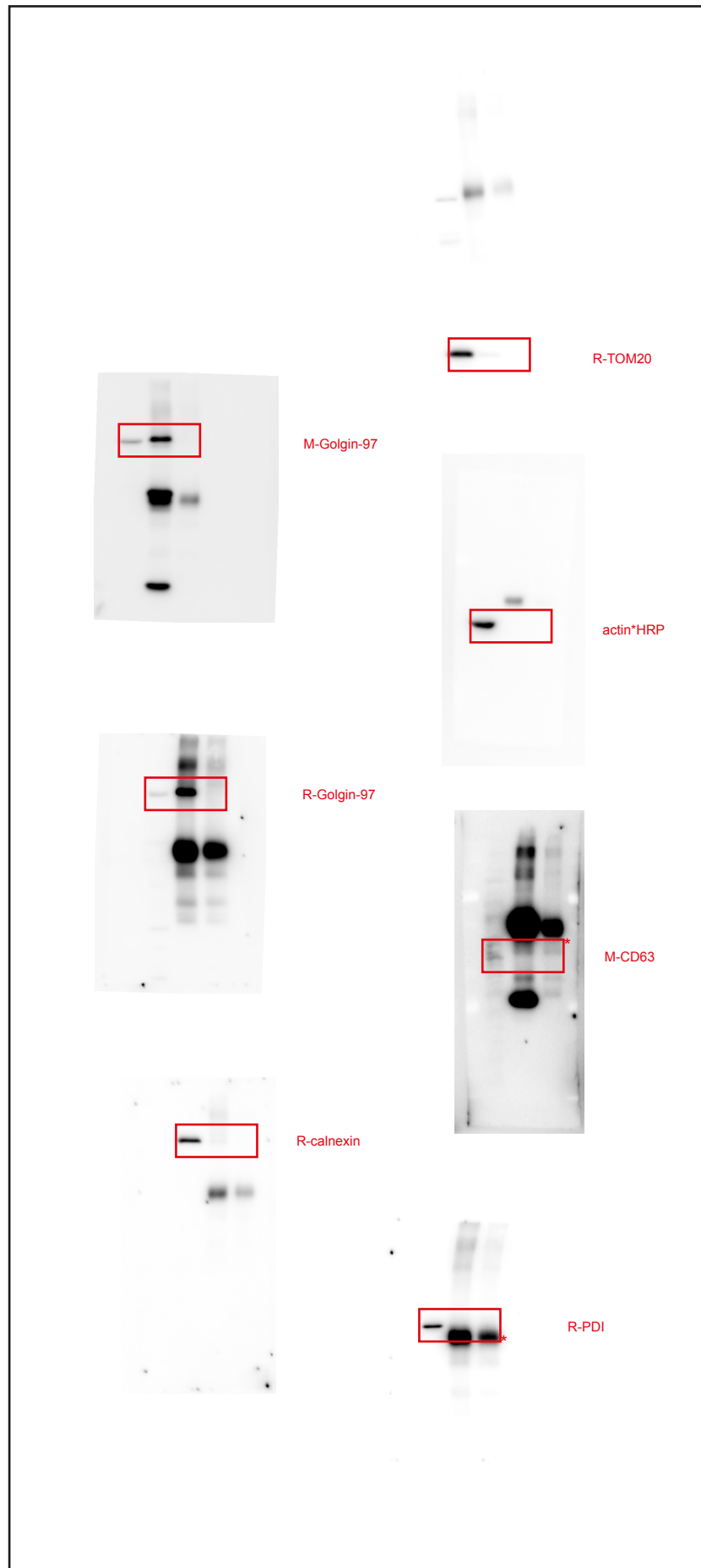

Supplement: Figure 7—source data 1. [file elife-91345-fig7-data1.zip › Figure 7_uncropped images/Figure 7-source data 01-uncropped_images.pdf]

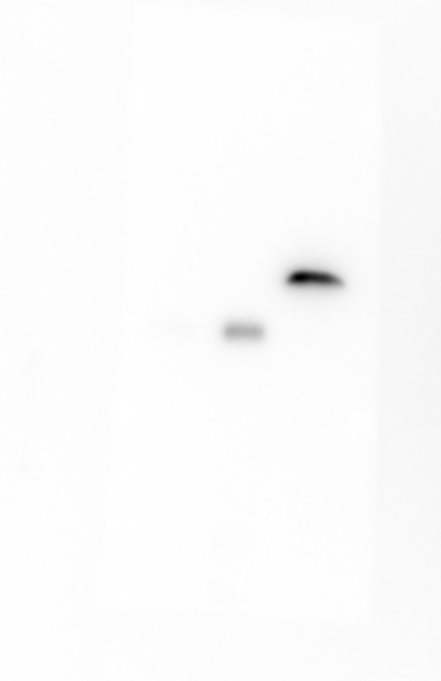

Supplement: Figure 7—source data 2. [file elife-91345-fig7-data2.zip › Figure 7-raw images/Figure 7-source data 05-Raw Images_actin.tif]

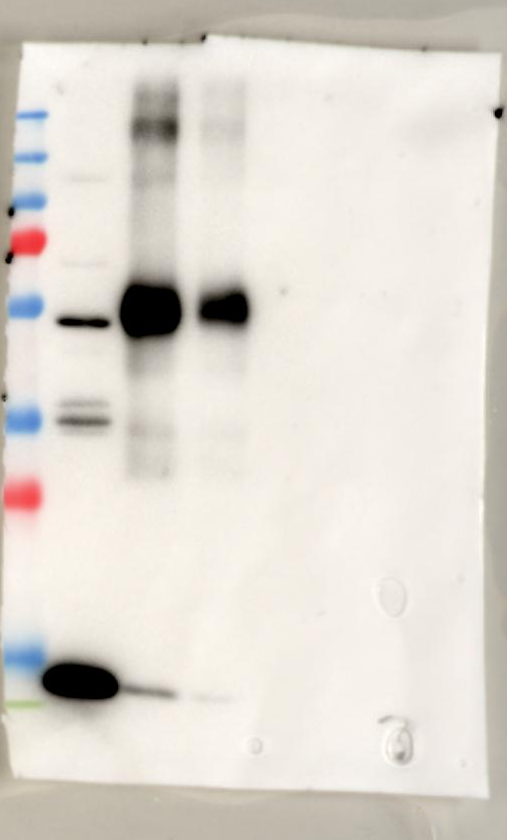

Supplement: Figure 7—source data 2. [file elife-91345-fig7-data2.zip › Figure 7-raw images/Figure 7-source data 11-Raw Images_TOM20.tif]

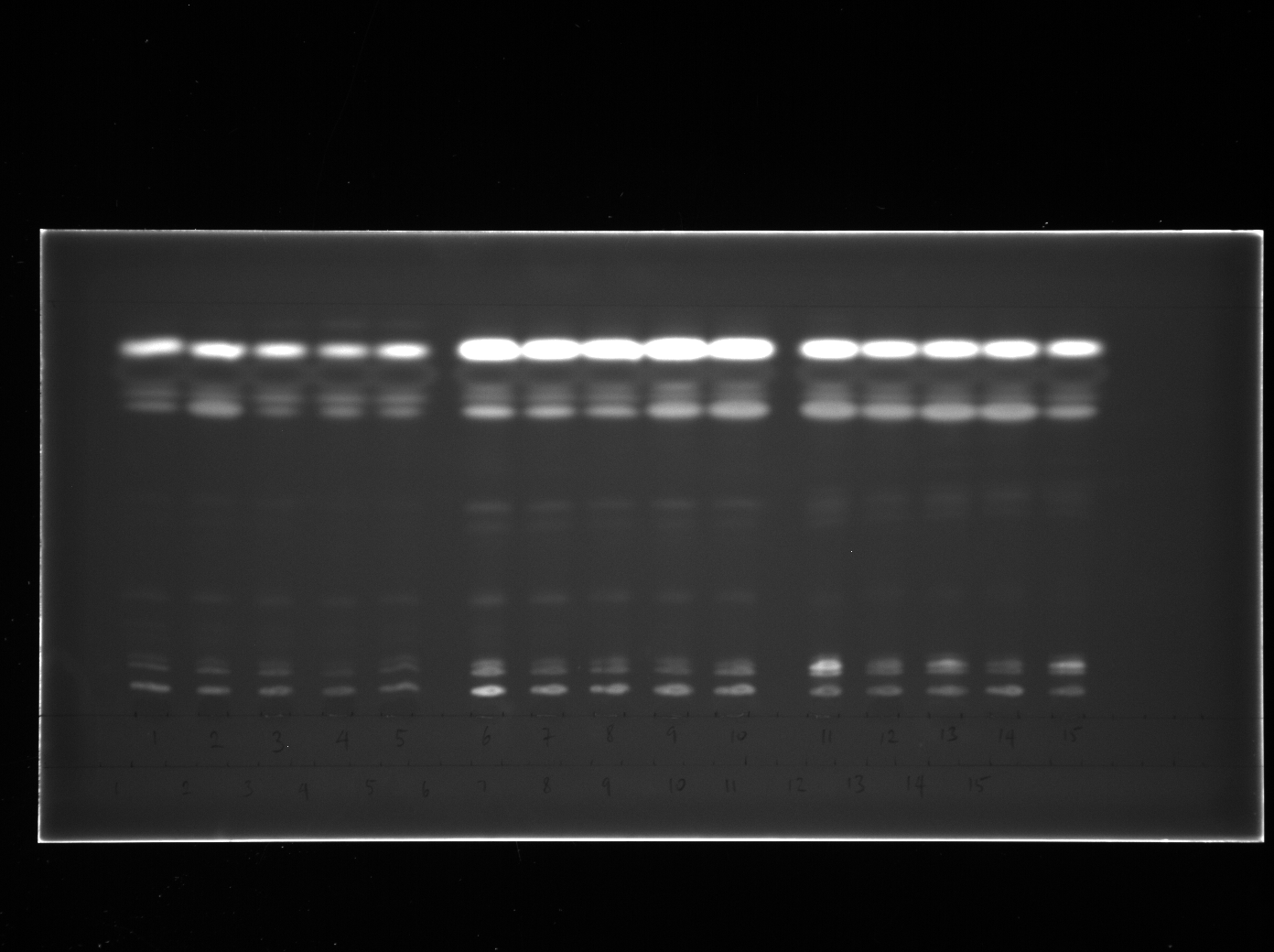

Supplement: Figure 7—source data 2. [file elife-91345-fig7-data2.zip › Figure 7-raw images/Figure 7-source data 03-Raw Images_TLC2.scn]

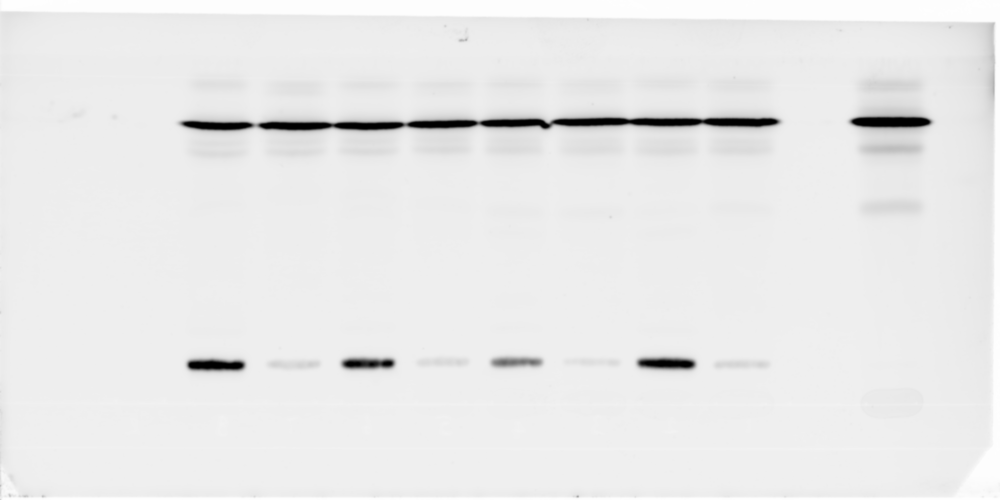

Supplement: Figure 7—source data 2. [file elife-91345-fig7-data2.zip › Figure 7-raw images/Figure 7-source data 04-Raw Images_TLC3.tif]

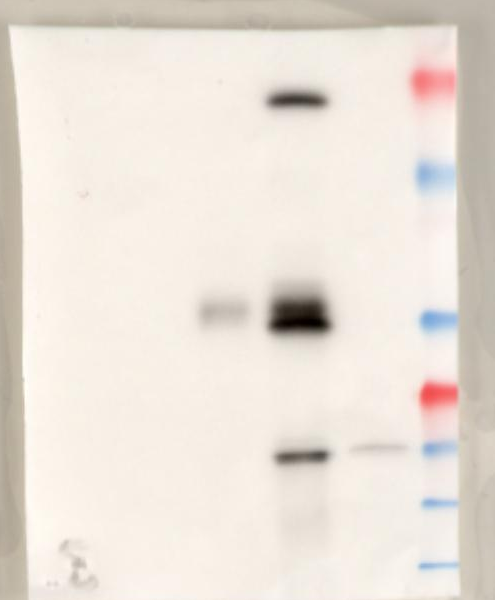

Supplement: Figure 7—source data 2. [file elife-91345-fig7-data2.zip › Figure 7-raw images/Figure 7-source data 08-Raw Images_M97.tif]

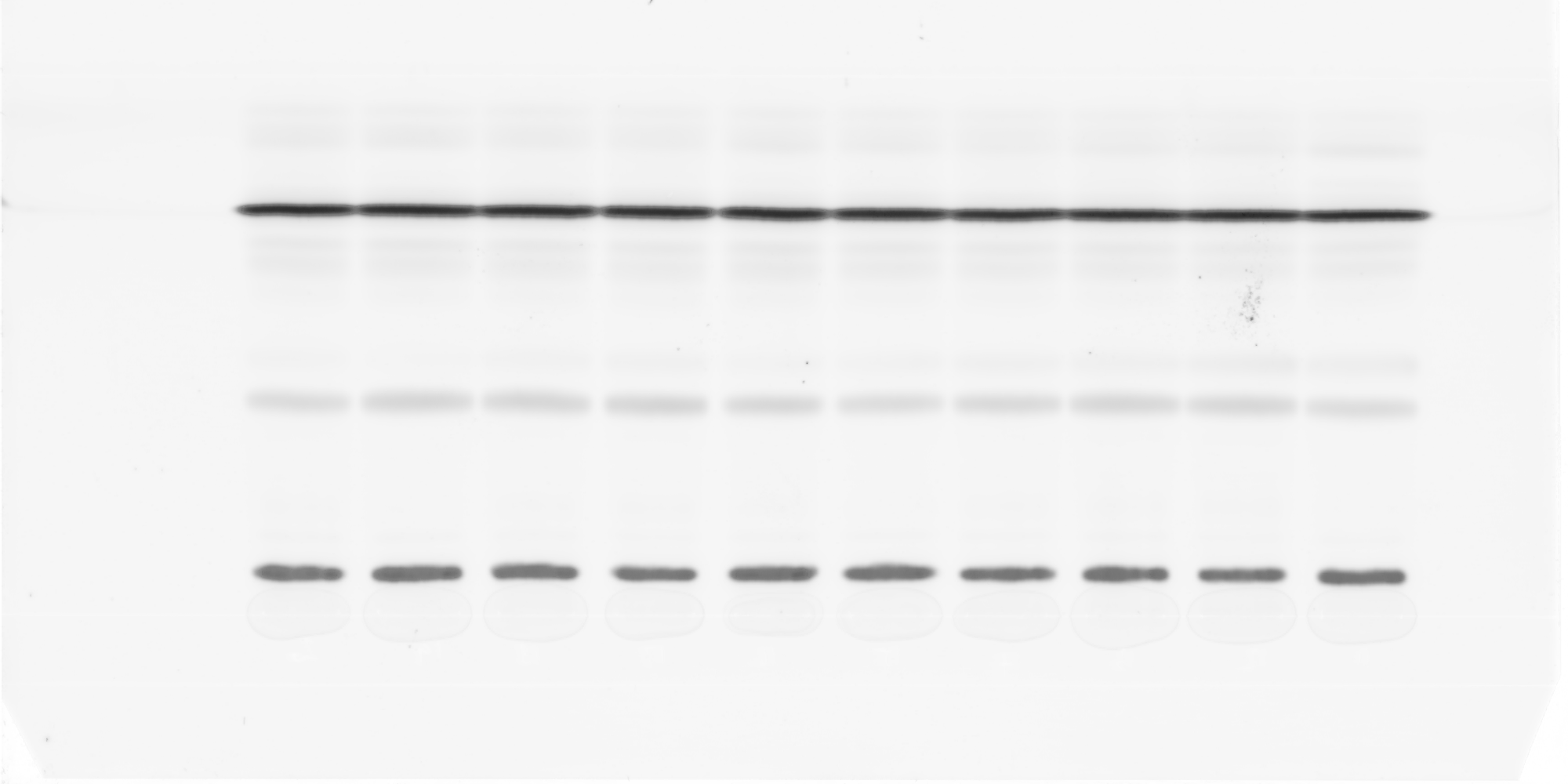

Supplement: Figure 7—source data 2. [file elife-91345-fig7-data2.zip › Figure 7-raw images/Figure 7-source data 02-Raw Images_TLC1.gel]

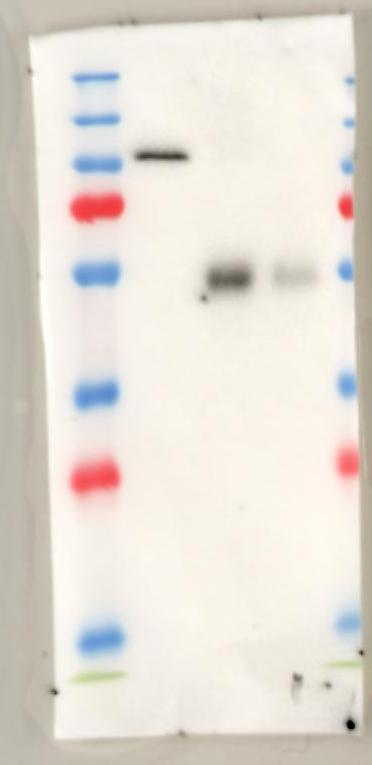

Supplement: Figure 7—source data 2. [file elife-91345-fig7-data2.zip › Figure 7-raw images/Figure 7-source data 07-Raw Images_CXN.tif]

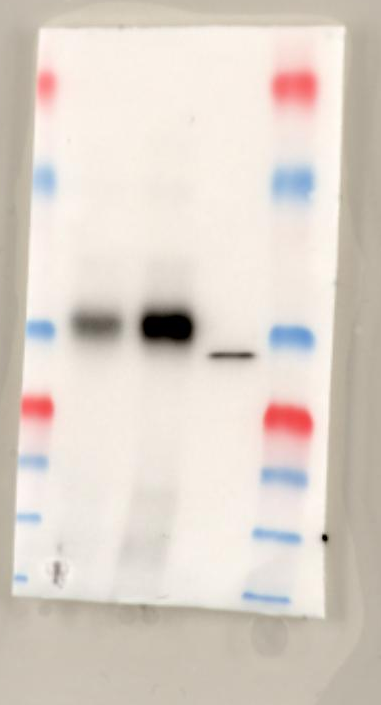

Supplement: Figure 7—source data 2. [file elife-91345-fig7-data2.zip › Figure 7-raw images/Figure 7-source data 09-Raw Images_PDI.tif]

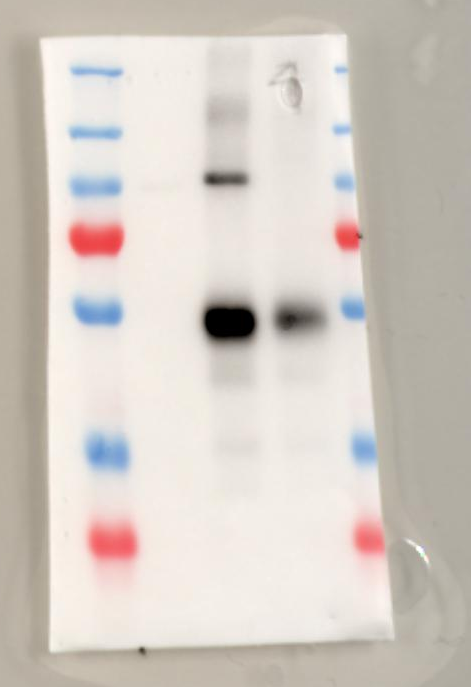

Supplement: Figure 7—source data 2. [file elife-91345-fig7-data2.zip › Figure 7-raw images/Figure 7-source data 10-Raw Images_RG97.tif]

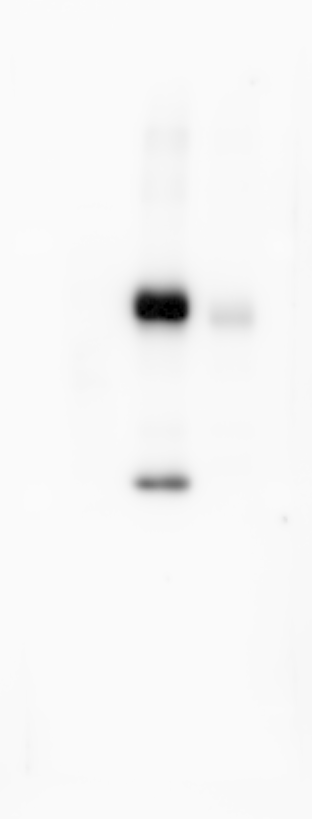

Supplement: Figure 7—source data 2. [file elife-91345-fig7-data2.zip › Figure 7-raw images/Figure 7-source data 06-Raw Images_CD63.tif]

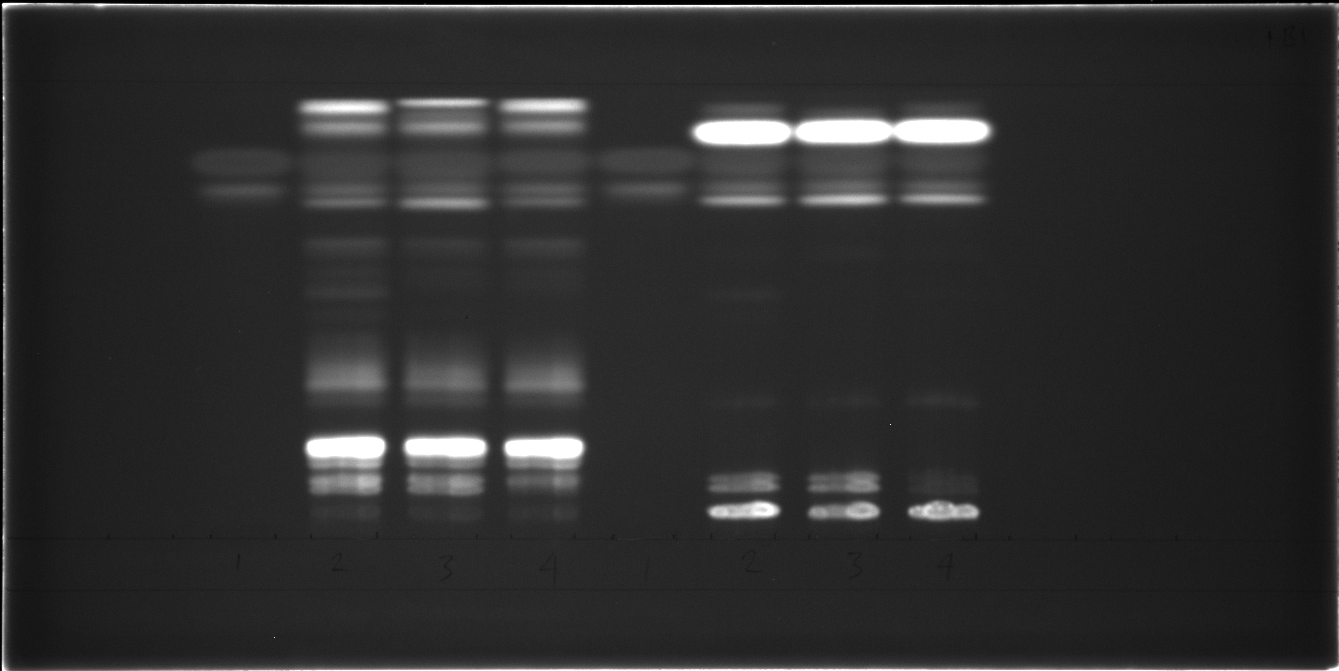

Supplement: Figure 7—figure supplement 1—source data 1. [file elife-91345-fig7-figsupp1-data1.zip › Figure 7-figure supplement 1-raw images/Figure 7-figure supplement 1-source data 2-Raw Images_TLC.tif]

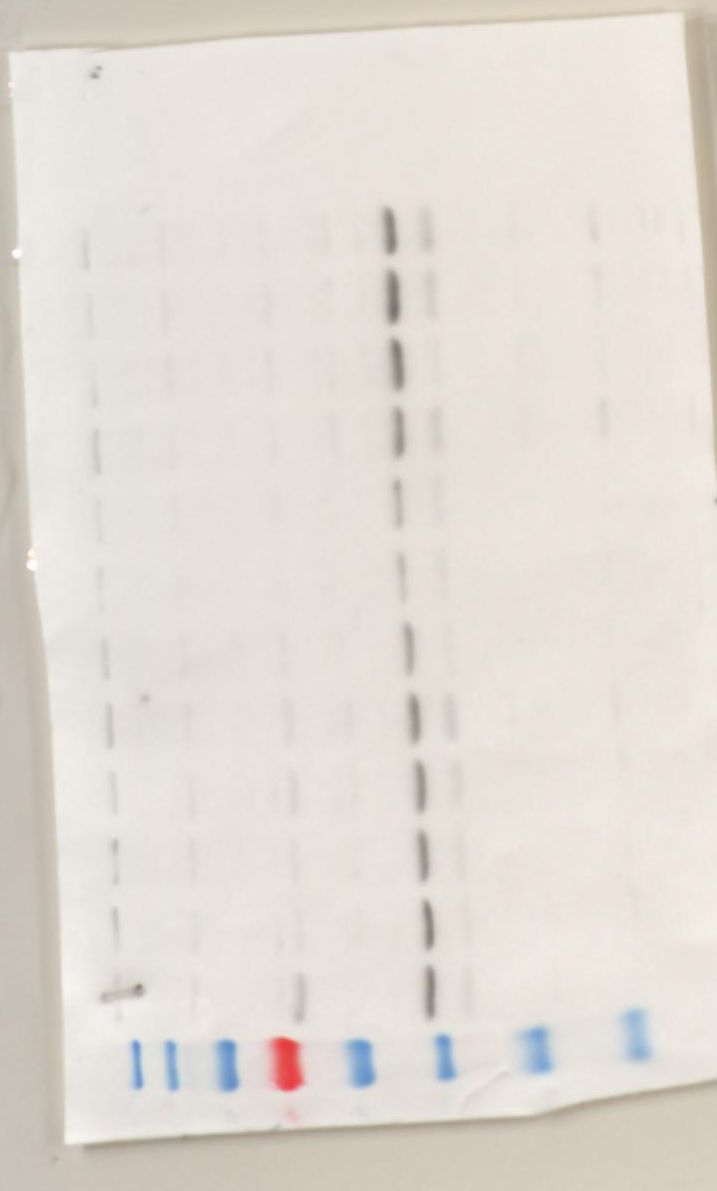

Supplement: Figure 7—figure supplement 1—source data 1. [file elife-91345-fig7-figsupp1-data1.zip › Figure 7-figure supplement 1-raw images/Figure 7-figure supplement 1-source data 3-Raw Images_a.tiff]

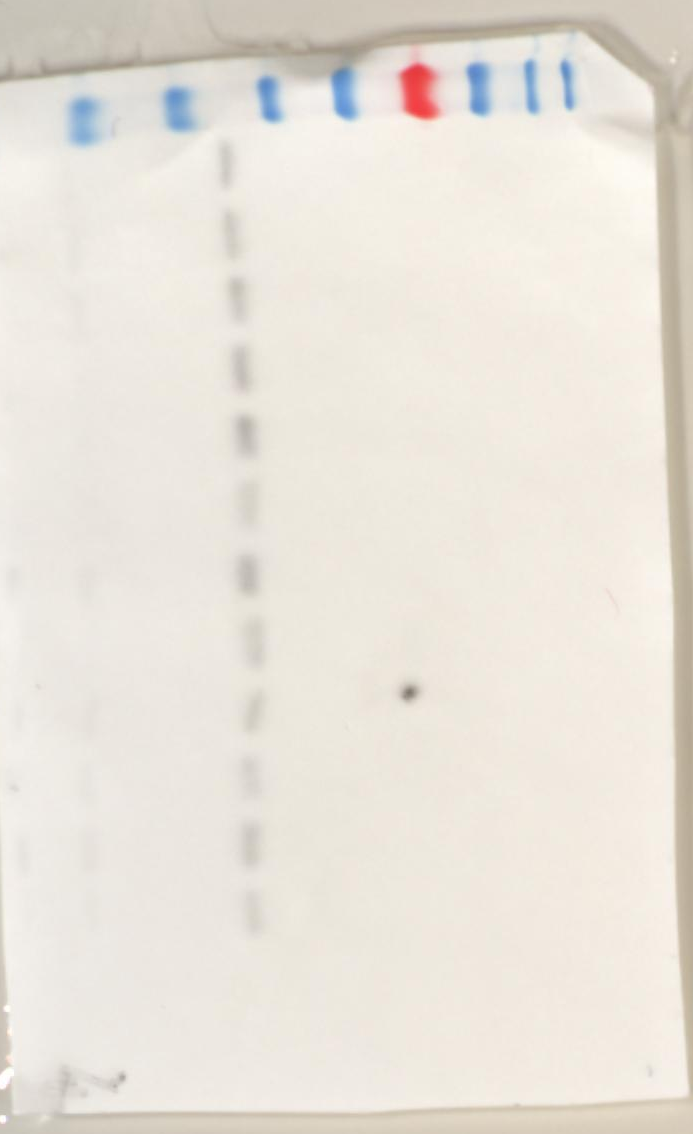

Supplement: Figure 7—figure supplement 1—source data 1. [file elife-91345-fig7-figsupp1-data1.zip › Figure 7-figure supplement 1-raw images/Figure 7-figure supplement 1-source data 4-Raw Images_b.tif]

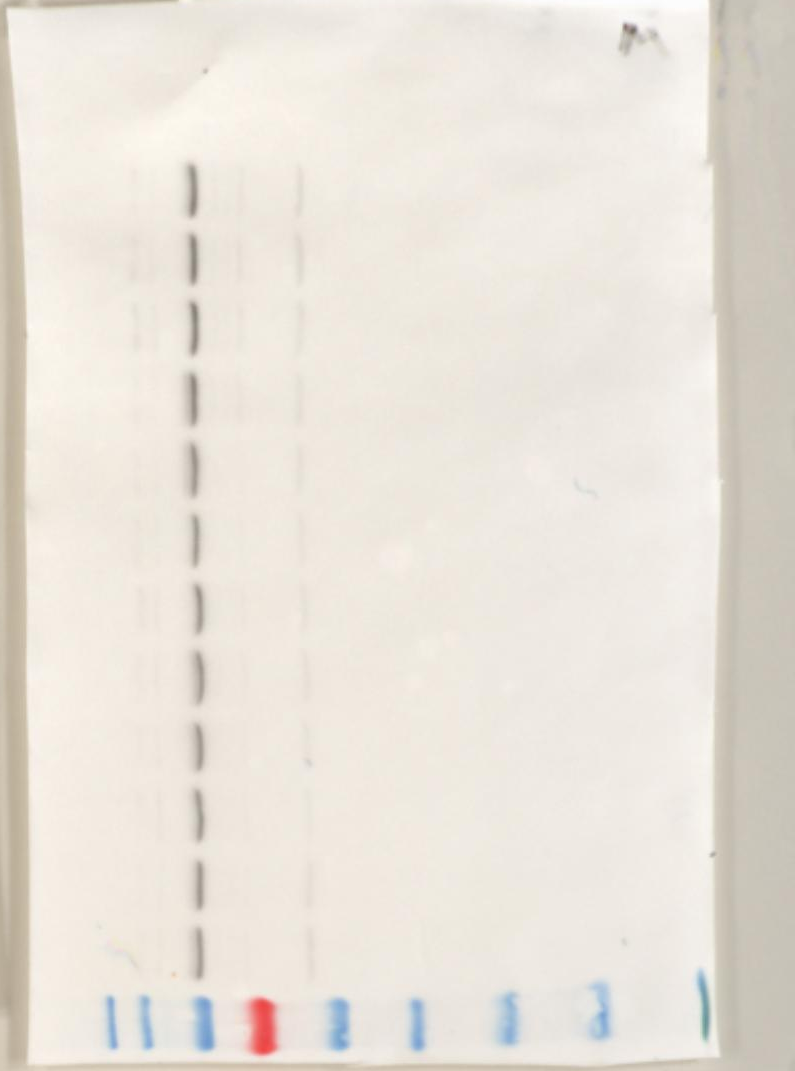

Supplement: Figure 7—figure supplement 1—source data 1. [file elife-91345-fig7-figsupp1-data1.zip › Figure 7-figure supplement 1-raw images/Figure 7-figure supplement 1-source data 5-Raw Images_c.tif]

Figure 7 – figure supplement 1

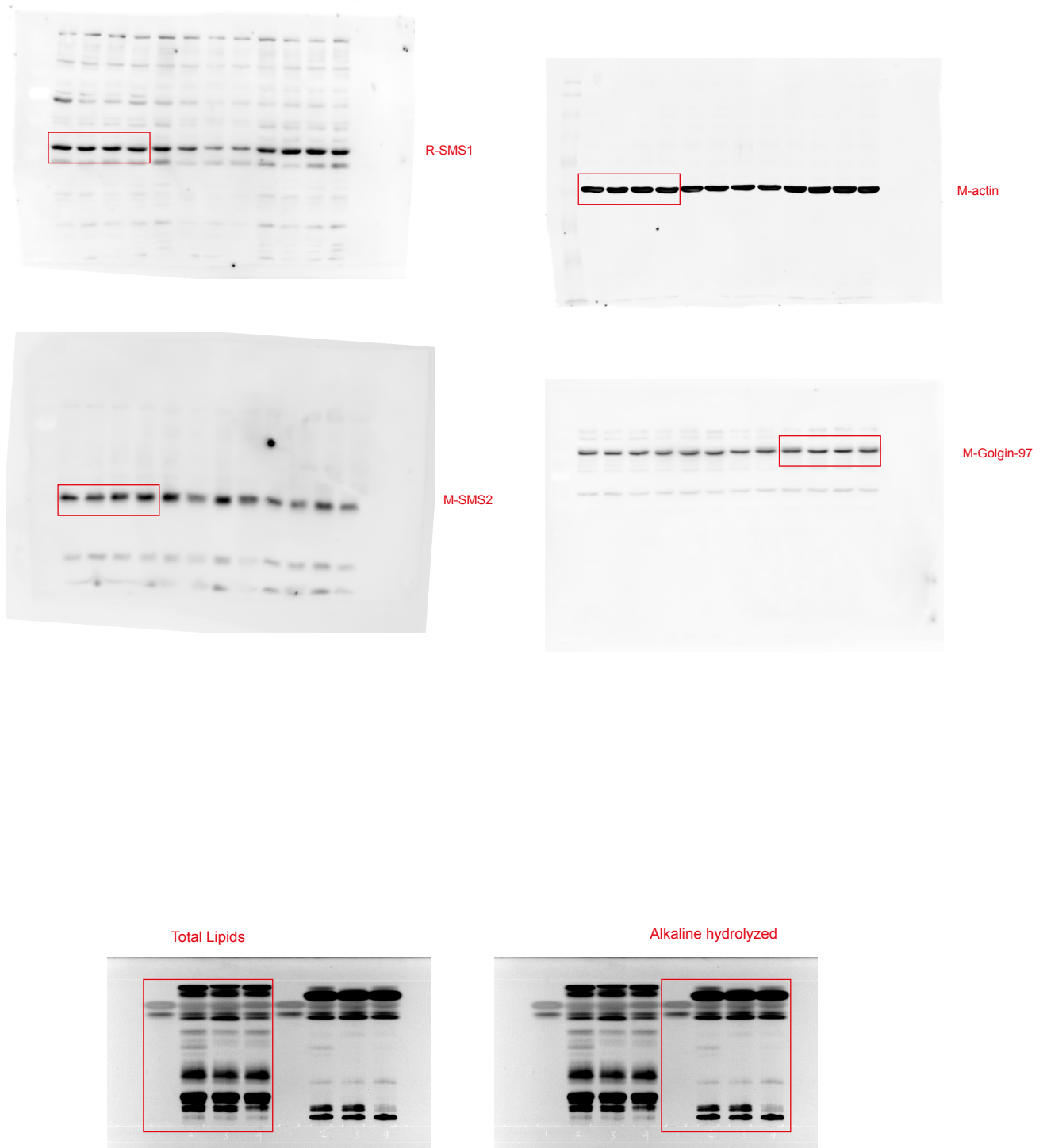

Supplement: Figure 7—figure supplement 1—source data 2. [file elife-91345-fig7-figsupp1-data2.zip › Figure 7-figure supplement 1-uncropped images/Figure 7-figure supplement 1-source data 1-uncropped_images.pdf]

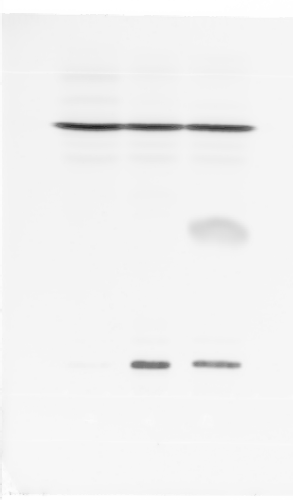

Supplement: Figure 7—figure supplement 2—source data 1. [file elife-91345-fig7-figsupp2-data1.zip › Figure 7-figure supplement 2-raw images/Figure 7-figure supplement 2-source data 3-Raw image files_TLC.tif]

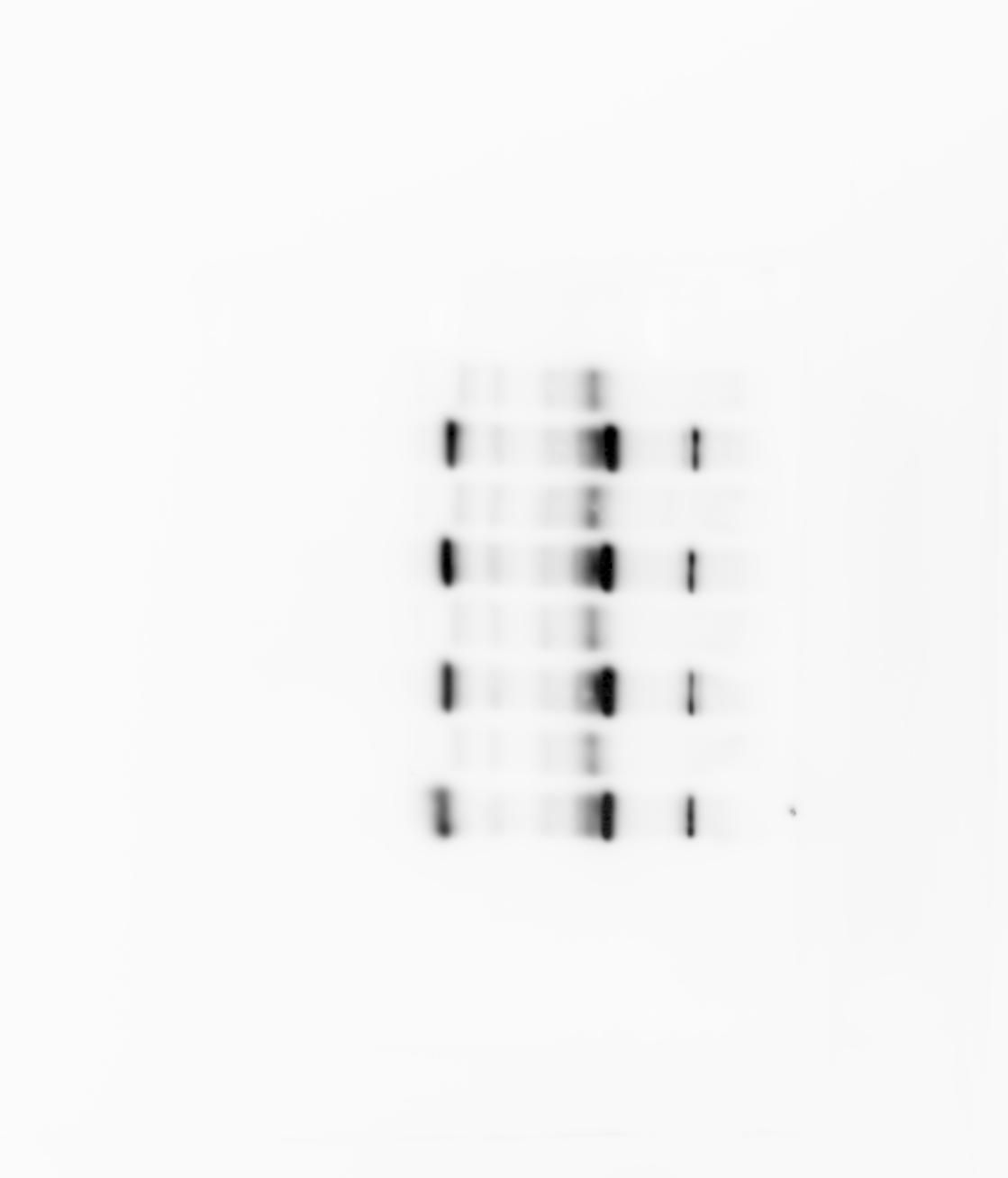

Supplement: Figure 7—figure supplement 2—source data 1. [file elife-91345-fig7-figsupp2-data1.zip › Figure 7-figure supplement 2-raw images/Figure 7-figure supplement 2-source data 2-Raw image files_blot.jpeg]

## Figure 7-figure supplement 2

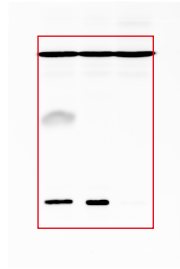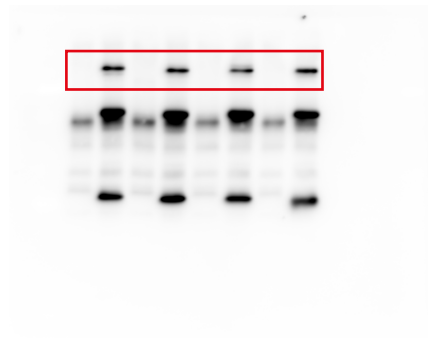

M-a-Golgin-97

Supplement: Figure 7—figure supplement 2—source data 2. [file elife-91345-fig7-figsupp2-data2.zip › Figure 7-figure supplement 2-uncropped images/Figure 7-figure supplement 2-source data 1.pdf]
